# Supplementary material for: Negative frequency‐dependent interactions can underlie phenotypic heterogeneity in a clonal microbial population
Source: Mol Syst Biol. 2016 Aug 3;12(8):877. doi: 10.15252/msb.20167033 (PMC5119493; doi:10.15252/msb.20167033)
Supplement: Supplementary file 1 — Appendix [file MSB-12-877-s001.docx]

**Negative frequency dependent interactions can underlie phenotypic heterogeneity in a clonal microbial population**

**Authors:** David Healey^1^, Kevin Axelrod^2^, and Jeff Gore^3^

^1^Department of Biology and ^3^Department of Physics, Massachusetts Institute of Technology, Cambridge, Massachusetts 02139, USA.

^2^Graduate Program in Biophysics, Harvard University, 400 Technology Square, NE 46-629, Cambridge, MA 02139

*Correspondence to: gore@mit.edu

**APPENDIX**

**Table of Contents**

**I**. Appendix Figures S1-S11 and Table S1 2-14

**II**. Didactic overview of environmental uncertainty vs negative frequency dependent selection (includes Appendix Figures S12-S15 and Table S2) 15-24

**III**. Computational modeling of a microbial foraging game (includes Appendix Figures S16-S22 and Table S3) 25-41

**IV**. References 42-43

**Author’s Note: About this document**

Section I of this document contains appendix figures 1 – 11, which will be most useful for experimentalists seeking a deeper understanding of the results of our experiments. In section II, we present a didactic overview of the distinction between two frameworks for understanding phenotypic heterogeneity of clonal populations in homogeneous environments: bet hedging and negative frequency dependent selection. This section will be most useful for readers lacking a background in game theory. In section III, we present the details of different mathematical models we have used to understand the results of our experiments.

**
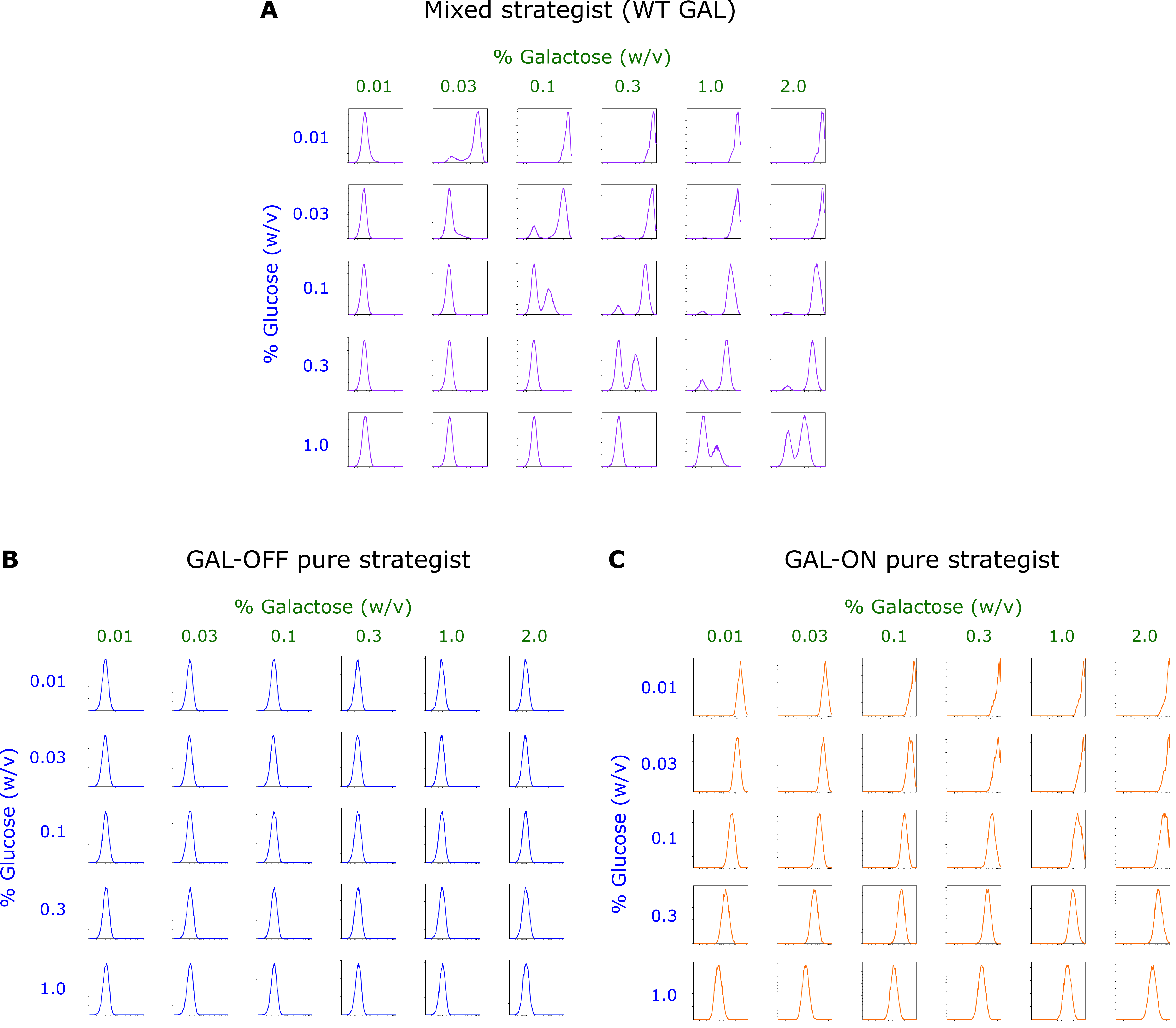
**

**Appendix Figure S1.** **Response of the GAL network to mixed glucose and galactose.** Wild type W303 mixed strategist (**A**), GAL-OFF pure strategist (**B**), and GAL-ON pure strategist (**C**) strains were induced in 1 μg/mL doxycycline and 0.01% glucose for 8 generations over a period of 24 hours, then diluted and incubated in 1 μg /mL doxycycline and various concentrations of glucose and galactose as shown. GAL activation states were measured after 8 hours via flow cytometry, and normalized histograms plotted against log YFP with a smoothing of 3 (horizontal scaling of plots is identical). Broadly speaking, the wild type W303 exhibits stable bimodal GAL expression over a wide range of roughly equal ratios of glucose and galactose concentration. (for in-depth characterization of the bimodal GAL response and its stability, see [[23](#_ENREF_23)] and [[25](#_ENREF_25)]. At higher ratios of glucose to galactose, it remains GAL-OFF, while at lower ratios it is unimodally GAL-ON. In all of the conditions wherein the wild type adopts a mixed strategy, the GAL-OFF pure strategist is inactivated, and the GAL-ON pure strategist is activated. However, in the cases of both the wild type and the GAL-ON pure strategist, increasing ratios of glucose to galactose result in lower activation levels of the GAL network.

**
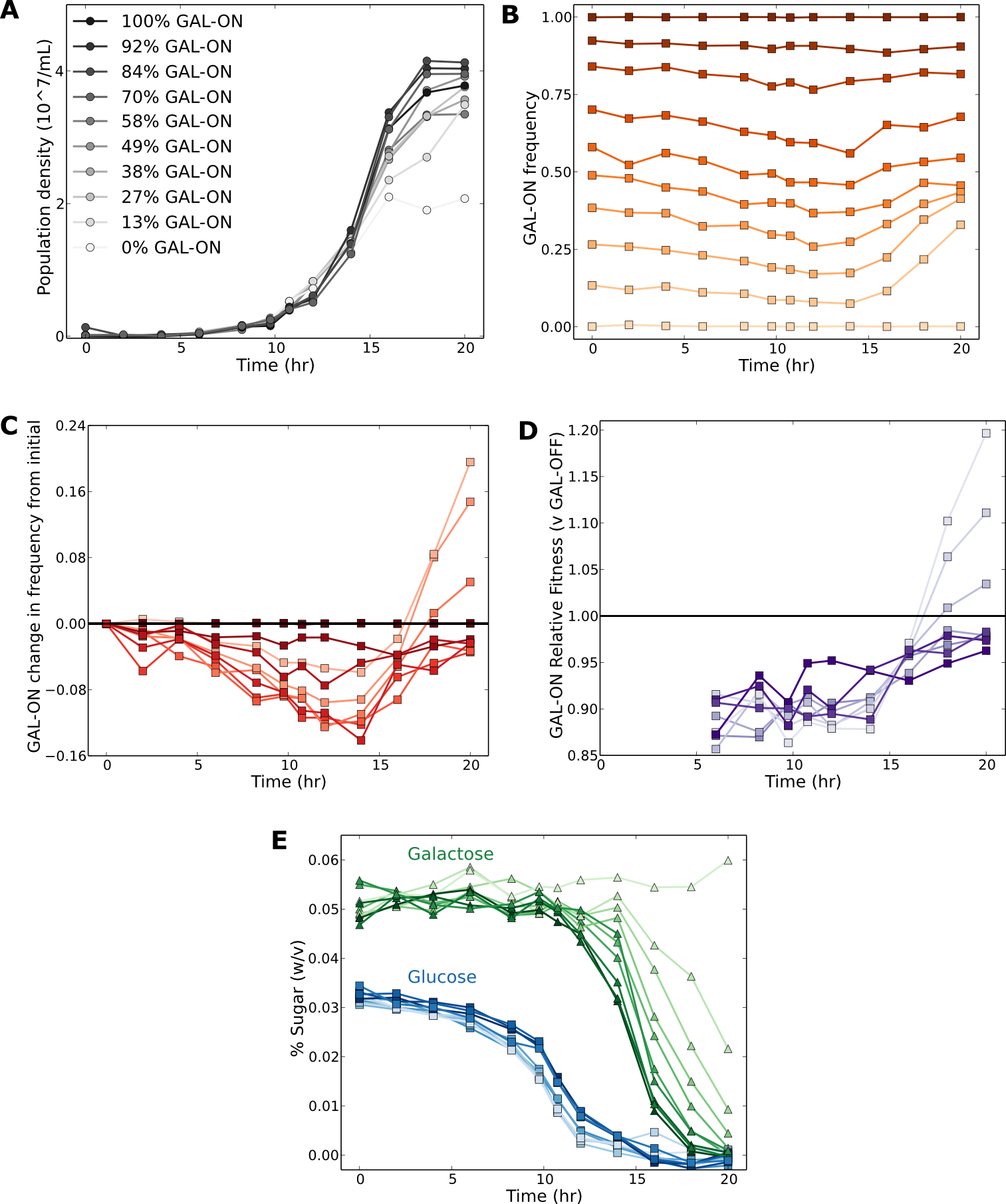
**

**Appendix Figure S2. Dynamics of growth, fitness, and sugar consumption for the 20-hr competition between GAL-ON and GAL-OFF pure strategists.**  **A,** population density is shown for yeast populations containing ten different initial fractions (from 0% to 100%) of GAL-ON over the course of a 20 hour incubation in the presence of 1 μg/mL doxycycline, 0.03% (w/v) glucose and 0.05% (w/v) galactose. **B,C,D** With the exception of the 0% and 100% GAL-ON populations, the fraction of GAL-ON initially decreases relative to GAL-OFF (with around a 10% relative fitness cost, **D**), until around the time glucose is depleted (**E),** at which point GAL-ON becomes more fit than GAL-OFF and increases in fraction. Final relative fitness of GAL-ON depends on initial fraction (**D**); low initial fractions of GAL-ON end at higher final fraction, while higher initial fractions of GAL-ON end at lower final fraction. In all plots, color gradients indicate the increasing initial frequencies of GAL-ON as described in panel A legend, except panel **D**, wherein the 100% and 0% initial GAL-ON populations are not shown because no relative fitness can be computed on a single strain. It should be noted also that in panel **C,** the 0% and 100% initial GAL-ON curves are shown, but are both overlayed on the x axis.


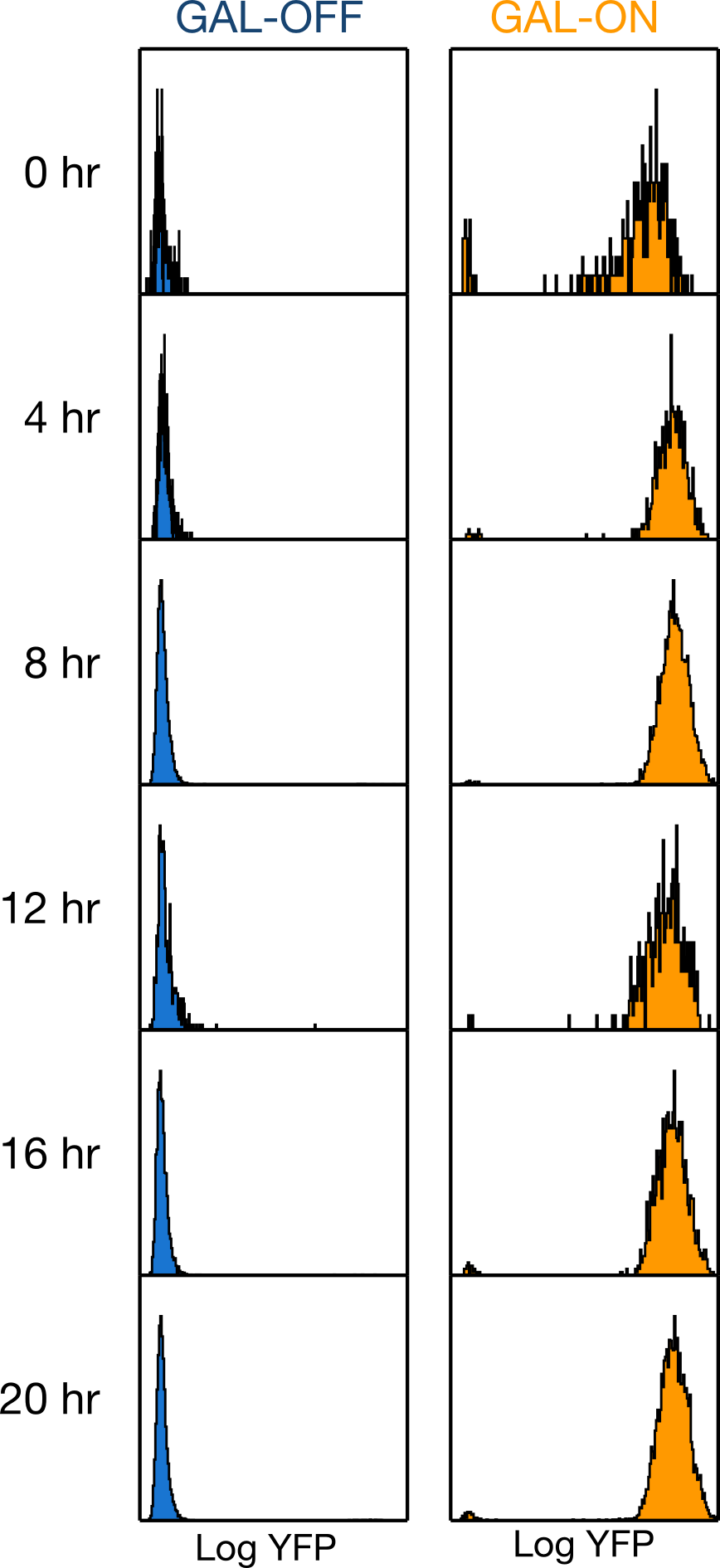


**Appendix Figure S3. Pure strategist GAL activation states remain stable over time.**

GAL activation histograms are shown over the course of the same 20 hr. competition described in Appendix Figure S2. Co-culture with an initial population GAL-ON frequency of 0.35 was grown overnight in the presence of 1ug/mL doxycycline and 0.01% glucose from an OD of 0.001 to a saturating density OD of 0.25. The culture was then diluted 1:1000 in 0.03% (w/v) glucose and 0.05% (w/v) galactose and incubated for 20 hours. GAL activation was measured throughout the course of the experiment via flow cytometry. During the course of the experiment, neither GAL-ON nor GAL-OFF deviate significantly. Comparison with Appendix Figure S4B and Figure 5 demonstrates that the GAL-OFF pure strategists are more stably inactivated than the GAL-inactive phenotype of the WT mixed strategist, which switches to GAL-ON upon depletion of glucose in the media.

**
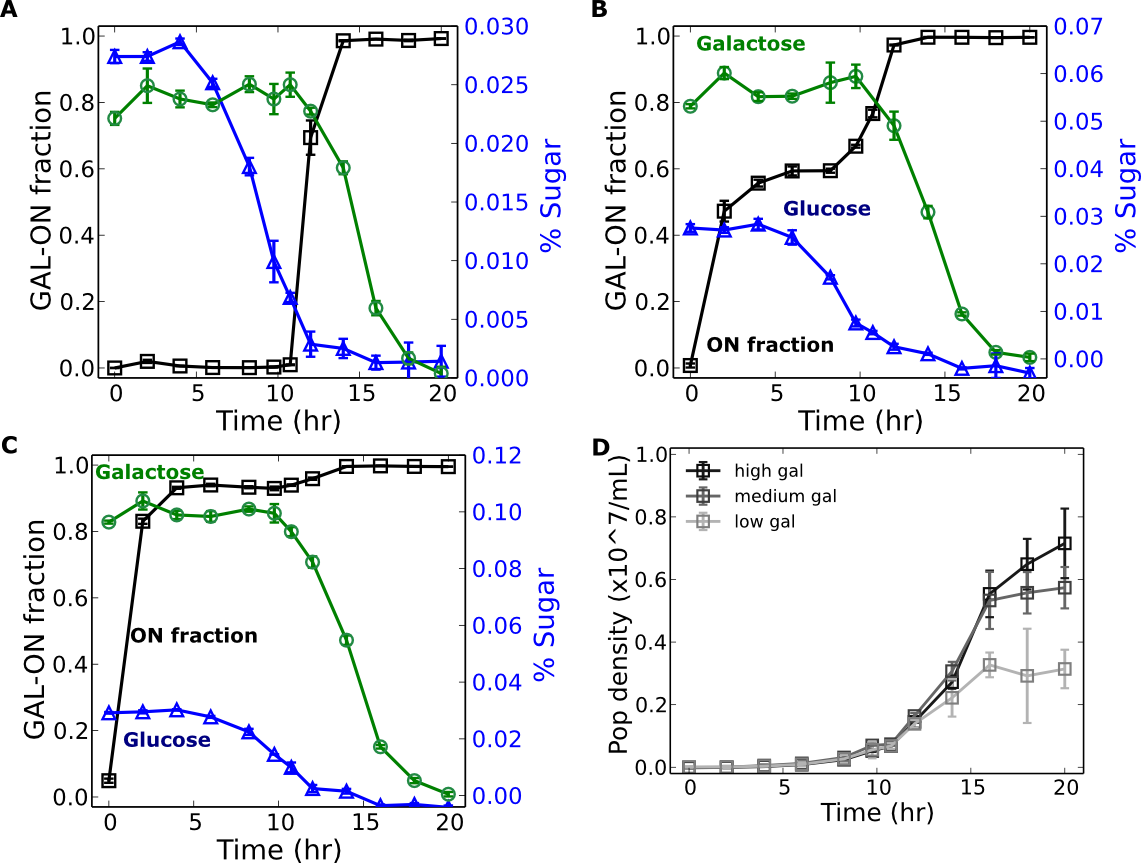
**

**Appendix Figure S4. Dynamics of wild type mixed strategist yeast in mixed glucose and galactose**. Yeast containing a wild-type GAL network were incubated in 0.03% (w/v) glucose and varying levels of galactose. **A,** In low (0.025%) galactose, the wild type remains unimodally off (black squares) until the glucose (blue triangles) is mostly consumed, then rapidly turns on its GAL network and consumes galactose (green circles). **B** At intermediate (.05%) galactose, the wild type exhibits bimodal expression of the GAL network (see Fig 4C) until the glucose is depleted, then rapidly switches to all ON. **C** At high galactose, the GAL network is rapidly activated in all cells. The 0-10 hr data in panels **B** and **C** suggest that even when the GAL network is activated in either half **(B)** or all **(C)** of the population, the yeast still consumes primarily glucose while it is still available. **D,** Population densities are shown for the three galactose conditions over the course of the 20 hour incubation.

**
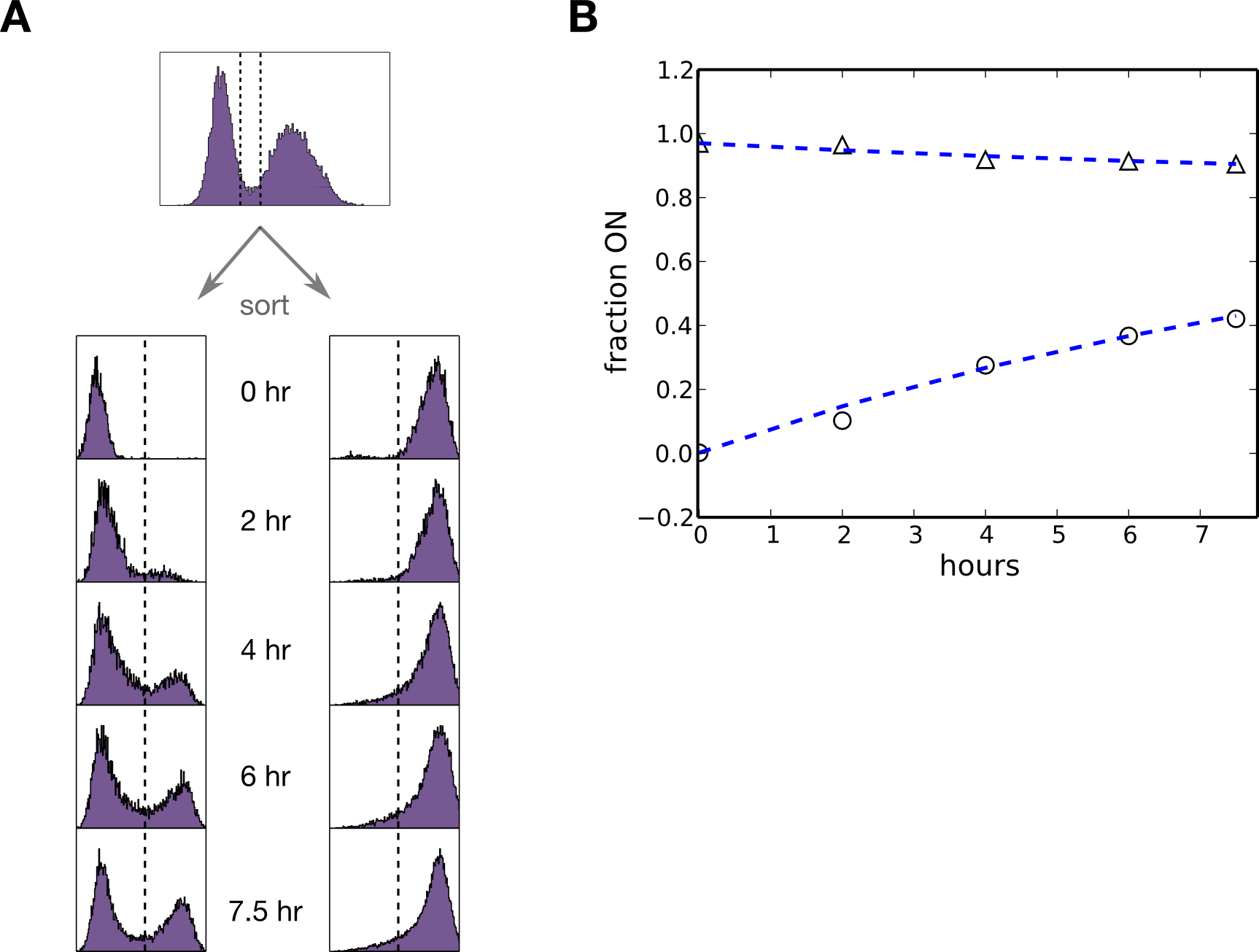
**

**Appendix Figure S5.**  **Stochastic switching of the wild type GAL network in mixed sugars.**  To measure the stochastic switching rate between the GAL-ON and GAL-OFF states for the wild type mixed strategist strain, we grew the RFP-labeled strain for 8 hours in 0.03% glucose and 0.05% galactose from a density of about $2\times{10}^{4}$ cells/mL. After 8 hours, cells were sorted by FACS into GAL-OFF and GAL-ON fractions, and diluted back into the original concentration of mixed sugars. **A)** Activation states for each of the sorted fractions are shown, as monitored by flow cytometry, over 7.5 hours after sorting. The switching rate in this condition was high enough to approximate the original distribution within about that time. **B)** Fraction GAL ON is plotted over time for both of the fractions shown in **A.** The OFF to ON and ON to OFF switching rates (K_ON_ and K_OFF_, respectively) were estimated to be K_ON_ = 0.08/hr, K_OFF_ = 0.015/hr via least squares fitting (dashed blue line.)

**
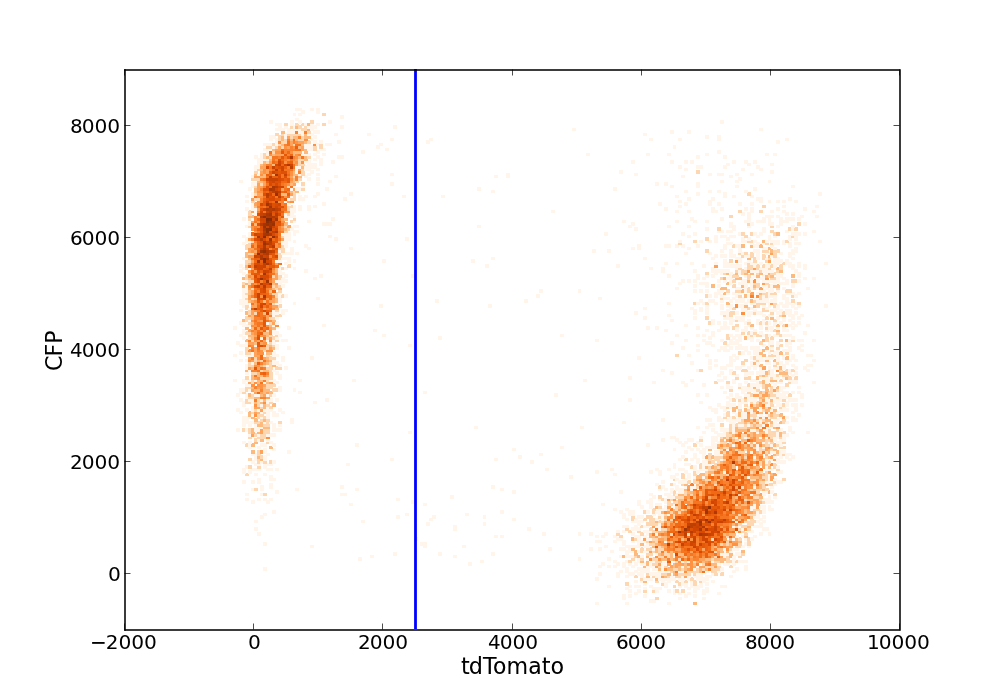
**

**Appendix Figure S6. Absolute and relative fitness determinations**

In order to calculate relative fitness, we first labeled each strain with either RFP (tdTomato) or CFP driven on a TEF1 constitutive promoter. We determined population frequencies before and after competition ( *f_i_* and *f_f_* , respectively ) for each strain via flow cytometry using a Miltenyi MACSquant flow cytometer (20,000+ cells per well). Separation of the two strains was very clean. Absolute fitness for each strain was calculated as the number of doublings:

Relative Fitness for each strain is calculated as follows:

where *f_i_* and *f_f_* are the initial and final fractions of the strain in the population, and *OD_i_* and *OD_f_* are the initial and final population densities as measured by absorbance at 600 nm in a microplate spectrophotometer.


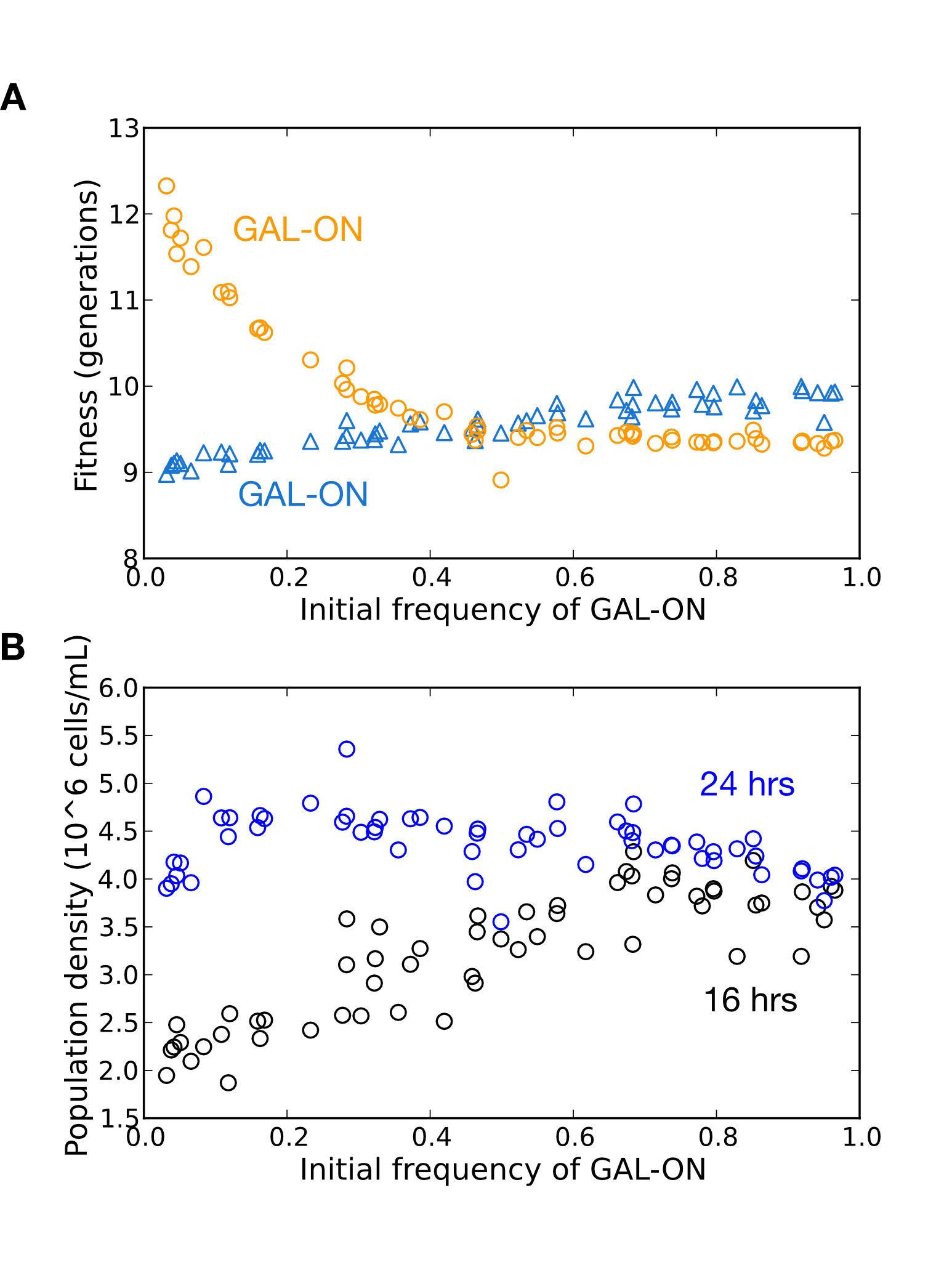


**Appendix Figure S7 High initial GAL-ON populations grow to saturating density faster than intermediate and low initial GAL-ON**

**A)** Fitness (in number of generations) is shown for 60 different initial starting GAL-ON fractions, in the same conditions described in main text figure 2D, but after 24 hours of growth rather than 20 hours. **B)** Total population growth is shown for those 60 initial starting GAL-ON fractions at 16 hours (black) and 24 hours (blue). By sixteen hours, populations of high initial GAL-ON have already reached very near saturating density, while populations with intermediate and low GAL-ON starting fractions are still growing. Showing saturating density at 24 hours reinforces the observation that the evolutionarily stable population composition is not growth optimal for the population. Comparison between panel A and Figure 2D demonstrates that the frequency dependent relative fitness of the two phenotypes does not qualitatively change between 20 and 24 hours.


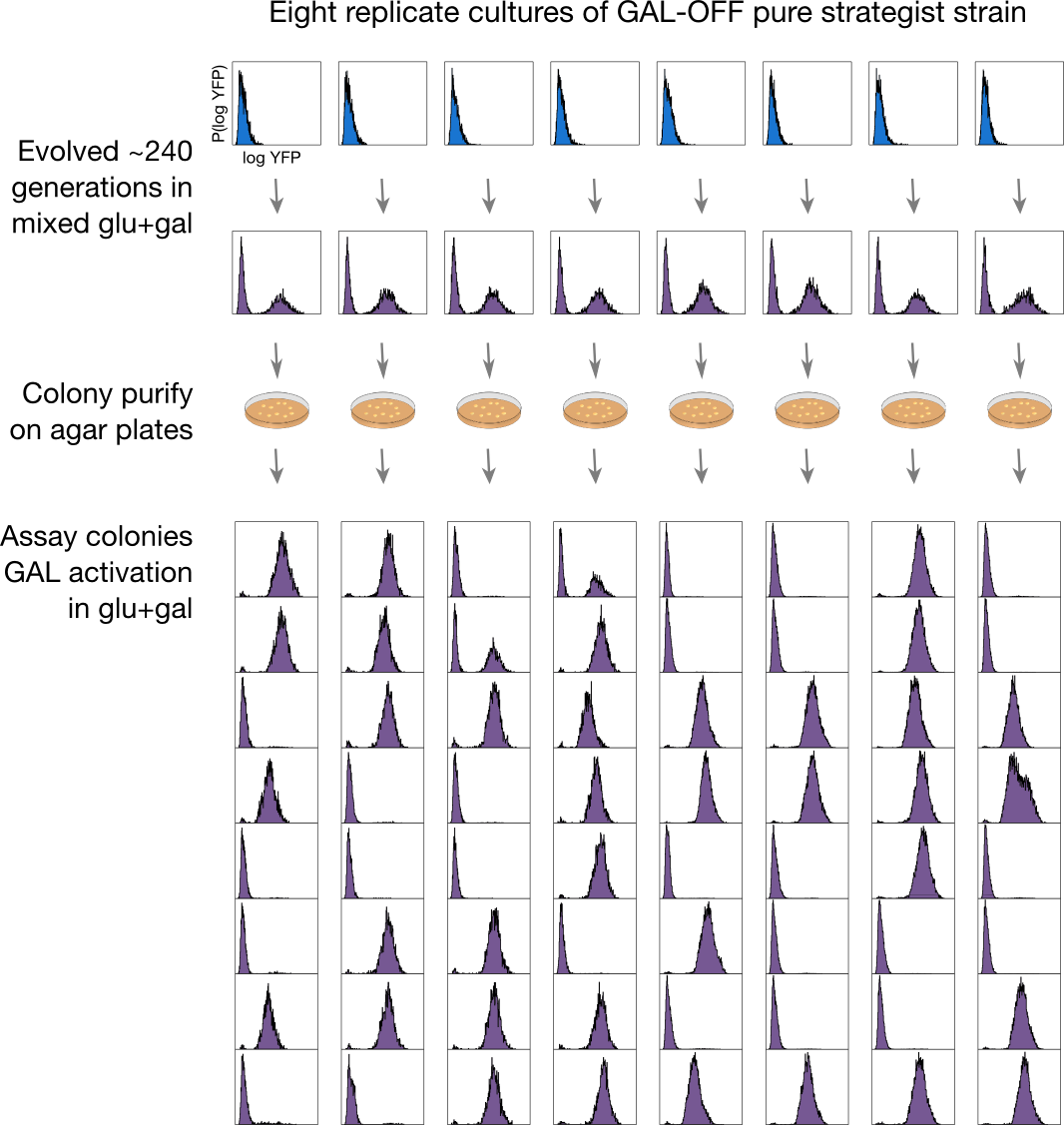


**Appendix Figure S8.** **Eight replicate evolved populations of GAL-OFF are dominated by coexistence between pure strategists**

Eight biological replicates of the GAL-OFF pure strategist strain (top row, blue) were incubated in the presence of doxycycline and 0.03% glucose and 0.05% galactose. Cultures were diluted 1000x daily into fresh media after reaching saturation for ~240 generations. During this time, each of the eight replicates evolved to a stable population mix of GAL-ON and GAL-OFF(see Fig. 5 for evolutionary history). To determine the composition of the evolved mixed population, cultures were plated on agar and eight individual colonies were grown separately in 0.03% glu and 0.05% gal (lower panel, columns). Each of the populations were predominantly composed of pure ON and pure OFF strains, indicating the evolution of a stable coexistence.

**
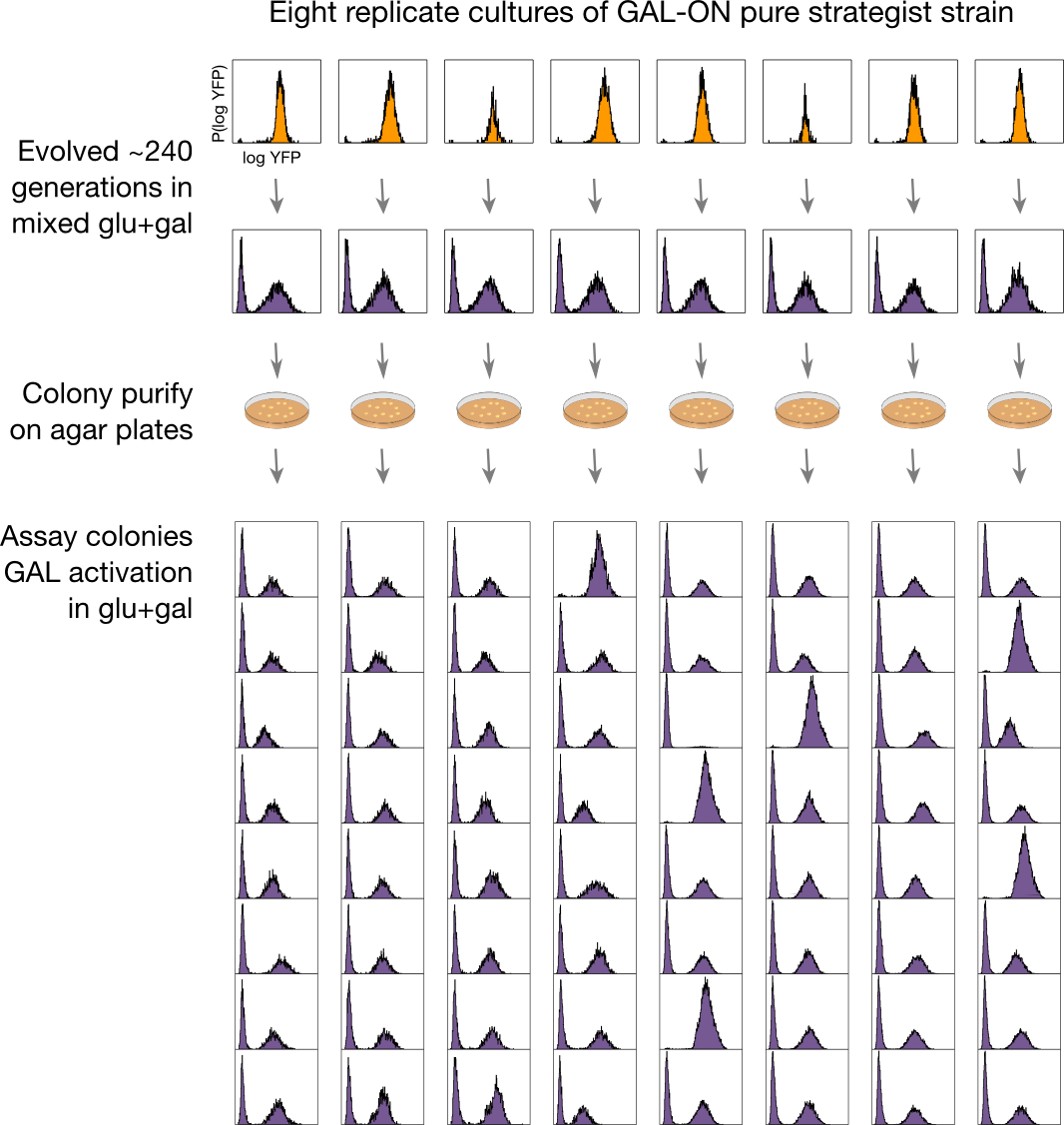
**

**Appendix Figure S9.** **Eight replicate evolved populations of GAL-ON are dominated by mixed strategists**

Eight biological replicates of the GAL-ON pure strategist strain (top row, orange) were incubated in the presence of doxycycline and 0.03% glucose and 0.05% galactose. Cultures were diluted 1000x daily into fresh media after reaching saturation for ~240 generations. During this time, each of the eight replicates evolved to a stable heterogeneous mix of phenotypes (see Fig. 5 bottom panel for evolutionary history). To determine the composition of the evolved mixed population, cultures were plated on agar and eight individual colonies were grown separately in 0.03% glu and 0.05% gal (lower panel, columns). This assay revealed that each of the eight populations was dominated by mixed strategist strains rather than a coexistence of pure strategists.


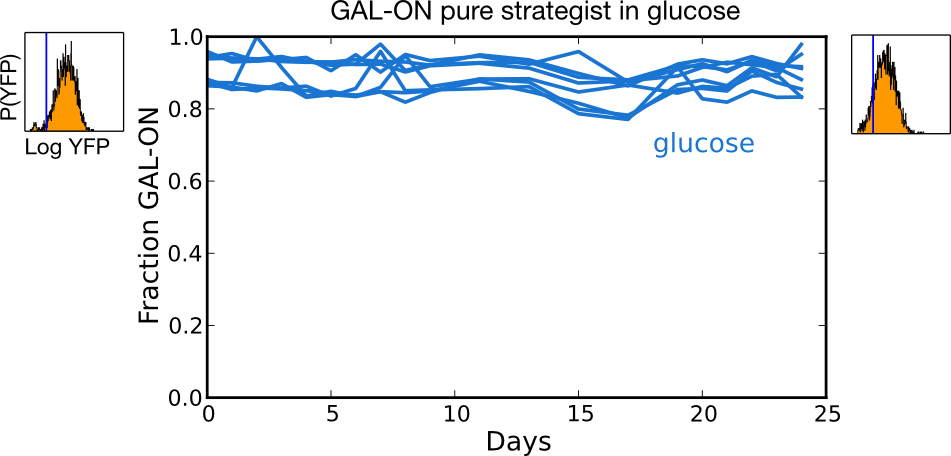


**Appendix Figure S10.** **Evolution of GAL-ON pure strategist in 0.1% glucose**

GAL activation histograms (orange) are shown for day zero (left) and day 24 (right) for the GAL-ON pure strategist in 0.1% glucose. This strain in this condition is peculiar in that its GAL activation state does not fall cleanly into the same ON or OFF category as do the other strains in the other conditions. The log YFP cutoff used to determine GAL activation throughout this work is shown as a vertical blue line in the histogram. Using this cutoff, the evolution of GAL-ON in just glucose is shown for all eight biological replicates; however, 85% ON in this case does not indicate bimodal activation as in the other intermediate cases shown in Fig. 5. In any case, there appears to be little systematic change over the course of the evolution experiment.


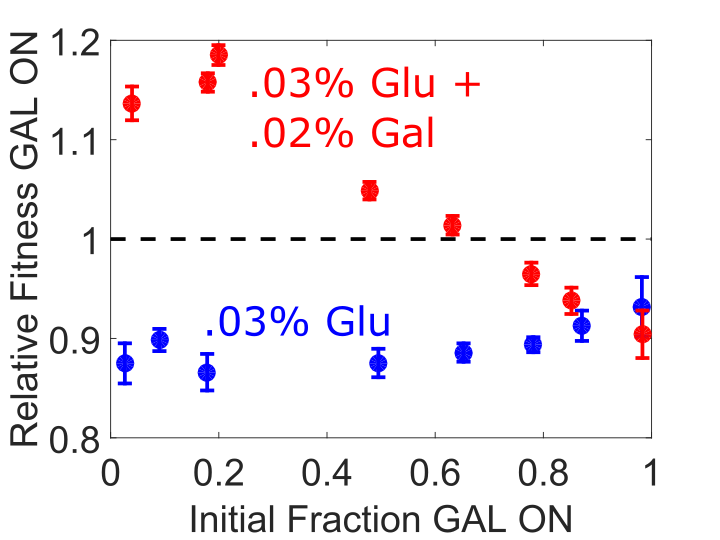


**Appendix Figure S11: Pure strategists show negative frequency dependence under quasi-continuous culture conditions in mixed sugar environment but not pure sugar environment**. Pure strategists were mixed in a range of initial fractions in .01% glucose for 24 hours. They were then grown to saturation for 17 hours in either pure glucose (blue) or a mixed sugar environment (red). At t=0, 3, 6, and 9 hours, the cells were diluted 2X to replenish with fresh sugars (blue: 0.03% glucose, red: 0.03% glucose + 0.02% galactose). At t = 0, 3, 6, 9, and 12 hours, the fractions of the two strains were measured using flow cytometry. Relative fitness of the two strains is computed using the relative fraction of the two strains at t=0 and t=12 hours. The relative fitness of the galactose strategist is plotted as a function of its initial fraction. Error bars were determined using bootstrap.

| Strain | Genotype |
| --- | --- |
| WT-R  (mixed strategist) | *MAT***a**/α, *ura*3/*URA*3*-*P*TEF1-tdTomato, his3*::*HIS*3/*his*3, *ade*2/*ade*2::*ADE*2-P*GAL1-YFP* |
| WT-C  (mixed strategist) | *MAT***a**/α, ura3/URA3-P*TEF1*his3::*HIS*3/*his*3, *ade*2/*ade*2::*ADE*2-P*GAL1-YFP* |
| Gal80i-R  (GAL-OFF) | *MAT***a**/α, *URA3-*P*TEF1*-*tdTomato*/*ura*3::*URA*3-P*TETO2-GAL80*, *his*3::*HIS*3/*his*3, *ade*2::*ADE*2- P*MYO2*-*rtTA/ade*2::*ADE*2-P*GAL1-YFP, gal80*∆::*KanMX*/*gal80*∆::*KanMX* |
| Gal80i-C  (GAL-OFF) | *MAT***a**/α, *URA3-*P*TEF1*-*CFP* /*ura*3::*URA*3-P*TETO2-GAL80*, *his*3::*HIS*3/*his*3, *ade*2::*ADE*2- P*MYO2*-*rtTA/ade*2::*ADE*2-P*GAL1-YFP, gal80*∆::*KanMX*/*gal80*∆::*KanMX* |
| GAL-ON (CFP) | *MAT***a**/α, *URA3*-P*TEF1-CFP*/*ura*3::*URA*3-P*TETO2-GAL3*, *his*3::*HIS*3/*his*3, *ade*2::*ADE*2- P*MYO2*-*rtTA/ade*2::*ADE*2-P*GAL1-YFP, gal3*∆::*KanMX*/*gal3*∆::*KanMX* |
| GAL-ON (CFP) | *MAT***a**/α, *URA3*-P*TEF1-CFP*/*ura*3::*URA*3-P*TETO2-GAL3*, *his*3::*HIS*3/*his*3, *ade*2::*ADE*2- P*MYO2*-*rtTA/ade*2::*ADE*2-P*GAL1-YFP, gal3*∆::*KanMX*/*gal3*∆::*KanMX* |

**Appendix Table S1. Yeast strains used in this study**

**Appendix Text S1: Environmental uncertainty and negative frequency dependence: an extended comparison**

**I. Introduction**

In this supporting text, we will explore the models and predictions of two hypotheses of for clonal phenotypic heterogeneity: bet-hedging in environmental uncertainty and mixed ESS as a response to negative frequency dependence. By understanding mathematically why each of these hypotheses predict the evolution of phenotypic heterogeneity, it will become more clear how to distinguish between them. The three main predictions of the “negative frequency dependence” hypothesis that differentiate it with bet-hedging are:

1. Mutual invasibility of phenotypes
2. Non-optimal stable evolutionary fixed points
3. Equal fitness across all phenotypes at the fixed point.

We will begin by examining uncertain environments, then examine negative frequency dependence, then end by illustrating the three predictions listed above.

**II. Unpredictable environments and evolutionary bet-hedging**

One reason why an organism may not benefit from completely deterministic decision-making is that the environment may be somewhat uncertain, and organisms may not have the time or ability to respond to sudden (and potentially catastrophic) shifts. They may therefore benefit from “hedging their bets” by adopting a phenotype that is suboptimal for their current environment, but which may increase their overall chances of survival in some possible future environment [[1-4](#_ENREF_1)]. The old investment adage about not putting “all your eggs in one basket” may just as well apply to microbial survival strategies as it does to finance.

Many examples of bet-hedging abound in the literature. Cohen [[5](#_ENREF_5),[6](#_ENREF_6)] initially developed the theory in the 1960s in the context of annual plants, which can either germinate, yielding more seeds in numbers that depend on environmental conditions, or remain dormant in the soil to germinate a different year. Dormant seeds decrease the plant’s yield during favorable years, but in unfavorable years, germinating plants may die while dormant seeds increase the chances for survival. The optimal strategy may therefore be for each seed to germinate sometimes and remain dormant sometimes [[7-10](#_ENREF_7)]. Ecologists have subsequently described bet-hedging strategies in a diverse range of plants and animals, in everything from body mass in wild boars [[11](#_ENREF_11)] to mating choice in Salmon [[12](#_ENREF_12)].

Some of the early examples of phenotypic heterogeneity in microbial populations resemble the plant germination problem and were candidates for being modeled as bet-hedging. For example, in multiple experimental bacterial systems it was observed that identical cells stochastically switch between fast-growing phenotypes and slow-growing “persister” cells or spores [[13](#_ENREF_13),[14](#_ENREF_14)], Persister cells and spores have much lower fitness in plentiful resources, but also have much higher survival of extreme environmental stress such as in the presence of antibiotics. Since that time, bet hedging has been used to explain scores of observed microbial phenotypic heterogeneity [[15-21](#_ENREF_15)]. It has become so popular as an explanation, however, that it is common for researchers to explain phenotypic heterogeneity as a response to environmental uncertainty with little empirical support and without considering alternative explanations (for review, see [[22](#_ENREF_22)]).

The defining concept of bet-hedging is that maximizing the geometric mean fitness of an individual over time often comes at the cost of lower arithmetic mean fitness [[1](#_ENREF_1),[7](#_ENREF_7),[8](#_ENREF_8)]. The geometric mean fitness is always less than the corresponding arithmetic mean by a function of the variance. Bet-hedging strategies maximize geometric mean fitness by reducing the fitness variation across time. It is important to note that this maximization of geometric mean is defined in the context where fitness is measured in discrete time as reproductive yield. However, in an exponentially growing population, where fitness is commonly defined as the exponential growth rate, then the arithmetic (not geometric) mean is optimized for populations growing in fluctuating environments.

A simple model of bet-hedging considers an organism with two possible phenotypes: *X* and *Y*. The organism exists in an environment that for each period of time can be in one of two states, which we will call Good (*G*) and Bad (*B*). The environment fluctuates between Good and Bad randomly, with the probability over time of Good environments denoted as *P_G,_* and bad as *P_B_ = (1-P_G_).*  The evolutionary yield, $Y_{i, j},$ is defined as the number of progeny produced by in individual of phenotype *i* in in environment *j* ). An individual’s fitness in environment *j*, *Wj*, is an average of the fitness of each phenotype in that environment, weighted by *f_i_*, the probability of the individual’s stochastically adopting phenotype *i*:

$$W_{j}= Y_{x,j}f_{x}+Y_{y,j}(1-f_{x})$$

As mentioned above, bet-hedging strategies maximize the geometric mean fitness of the individual across time. The geometric mean fitness :

$$\bar{W}={[Expected fitness in environment G]}^{P_{G}}{[ Expected fitness in environment B]}^{(1-P_{G})}$$

Combining the last two equations, yields the equation for geometric mean fitness:

$$\bar{W}={[Y_{x,G}f_{x}+Y_{y,G}(1-f_{x})]}^{P_{G}}{[ Y_{x,B}f_{x}+Y_{y,B}(1-f_{x})]}^{(1-P_{G})}$$

Solving for $f_{x}^{*}$, the probability of adopting phenotype x that maximizes geometric mean fitness, yields:

$$f_{x}^{*}=\frac{Y_{x,B}{Y_{y,G}P}_{G}-Y_{x,B}Y_{y,G}-Y_{x,G}{Y_{y,B}P}_{G}+Y_{y,G}Y_{y,B}}{\left( Y_{x,G}-Y_{y,G} \right) (Y_{x,B}-Y_{y,B})}$$

In a common example of bet-hedging (shown in the figure below), the phenotypes are to either grow (X) or remain dormant (Y). In a good environment, a growing phenotype produces another individual (*Y_x,g_ = 2*), while in a bad environment, the growing phenotype has a 90% chance of dying (*Y_x,b_ = 0.1*). Remaining in a dormant state results merely in the survival of the individual regardless of environment (*Y_y,g_ = Y_y,b_ = 1*). If good environments occur with a probability 0.8, then the optimal probability of adopting the growth phenotype would be about 0.69.


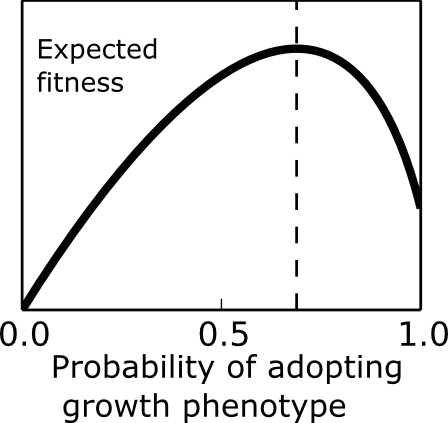


**Appendix Figure S12.** A simple example of bet-hedging. Geometric mean fitness for an individual seed is shown as a function of the probability that the seed will germinate vs remain dormant. In the example, good environments occur with probability 80%, and nondormant individuals yield one viable offspring in good times, while in times of stress, 90% of nondormant individuals die.

As is clear in the above figure, an individual’s fitness will be maximized in the long term by adopting a strategy of remaining nondormant with probability 0.69.

**III. Negative frequency dependence and evolutionarily stable mixed strategies (mixed ESS)**

Another reason that organisms would benefit from phenotypic heterogeneity is the concept of negative frequency dependent selection of phenotypes, whereby each phenotype’s fitness decreases as it becomes more prevalent in the population. A common case of negative frequency dependence is *mutual invasibility*, where each phenotype is more fit than the other when rare.

It is intuitively clear that when an environment causes such mutual invasibility of phenotypes, the result is likely to be a stable coexistence of phenotypes in the population. Generally we think of these stable points as genetic polymorphisms, or coexistences between strains or species. An isogenic population, however, can mimic the stability of a genetic polymorphism by adopting a strategy of phenotypic heterogeneity with the appropriate probabilities. In the field of evolutionary game theory, where the theory such strategies were first explored decades ago, this is called the evolutionarily stable mixed strategy, or mixed ESS.

Any situation of mutual invasibility is a candidate for mixed ESS, but the canonical example is the hawk-dove model of animal conflict, where animals can either be aggressive (hawk) or passive (dove) in fighting over some resource. The fitness of an individual depends on the composition of the population it is in. Note that Hawks and Doves are not intended to be separate species, but rather behavioral variants of the same species. There are three possible pairings in a symmetric two-player Hawk-Dove game:

1. If Hawk meets Dove *(H,D),* Dove flees and Hawk gets the resource (*V*). Dove retreats and receives a payoff of zero.
2. If Hawk meets Hawk *(H,H)* they fight. Each has a ½ probability of getting the resource, but only after each incurs an injury cost (*C*) greater than half the value of the resource.^[[1]](#footnote-1)^ Their expected payoff is ½ (*V-C*). The key point is that the expected payoff of Hawk against Hawk, *E(H, H),* is less than the payoff from retreating against Hawk (H,D).
3. If Dove meets Dove (*D,D*) they share the resource. Their expected payoff is ½ (*V*).

In matrix notation, the payoffs are as follows:

Appendix Table S2. The two-player hawk-dove game

|  |  | Player 2 | |
| --- | --- | --- | --- |
|  |  | Hawk | Dove |
| Player 1 | Hawk | (½*V - C*, ½*V - C)*) | (V, 0) |
|  | Dove | (0 , V) | (½ V , ½ V) |

Since the expected payoff of playing Hawk against Hawk, *E(H,H)* is less than the expected payoff of playing Dove against Hawk, it is apparent that the Hawk strategy cannot be evolutionarily stable. In a population of all Hawks, a single Dove will receive a higher payoff than the rest, and invade the population. Likewise Dove is also not evolutionarily stable, since against a Dove opponent, hawks can win the entire resource. *E(H,D)* > *E(D,D)*; a single Hawk will invade a population of all Doves. Thus, the pure strategies are mutually invasible. If we assume that all individuals play pure strategies (they must either be Hawks of Doves), then the stable result will be a coexistence of the two kinds of individuals.

Suppose now that individuals can implement a mixed strategy rather than being only Hawk or only Dove. We will define a mixed strategy as “play Hawk with probability *p*, and Dove with probability *(1-p)”*. The ESS of our hawk-dove game, P_ESS_ is found, not by optimizing the expected as in our bet-hedging example, but by finding the fraction of hawks and doves in a population that would result in *an equal expected payoff* for playing hawk and playing dove. For an individual in a population consisting of both Hawks and Doves, the expected payoff of adopting a strategy depends on the fraction of Hawks and Doves in a population. If Hawks exist with frequency *f* and Doves with frequency *(1-f),* then for any strategy *I*, the expected payoff of adopting *I, E(I,f)*, is a weighted average, given as:

*E(I,f) = f * E(I,H) + (1-f) * E(I,D)*

The evolutionarily stable mixed strategy will be to choose *p* such that *E(H) = E(D).* Since for a clonal population playing a mixed strategy, $p = f$, solving the above equation generally for E(D) = E(H) yields an evolutionary stable probability:

$$P^{ESS}=\frac{E\left( D,D \right)-E(H,D)}{E\left( H,H \right)-E\left( D,H \right)-E\left( H,D \right)+E(D,D)}$$

Substituting the payoffs in Table 1, we have:

$$P^{ESS}=\frac{V}{2C}$$

The key characteristic to note in this example is that evolution will favor equal payoffs rather than optimal payoffs, because only when the payoffs are equal for the phenotypes is there no incentive for any individual to switch to the other phenotype. For $V=\frac{2}{3}C$, for example, the optimal scenario—the one which provides the highest expected fitness—is for all individuals to be Doves all the time, while the mixed ESS is to play Hawk with frequency 1/3 (see Fig S13).

**Appendix Figure S13.** A simple hawk-dove game with $C=\frac{3}{2}V$. The ESS occurs when Hawks are 1/3 of the population, so by playing Hawk with frequency 1/3, a clonal population can be evolutionarily stable. Note also that the payoff for the population as a whole (below) is maximized when there are no Hawks, but that such a population is prone to invasion by Hawks. Thus, the growth-optimal frequency is not necessarily evolutionarily stable.

Another key characteristic of a mixed ESS like that, unlike a bet-hedging strategy, the evolutionary fixed point is not the one that maximizes the expected fitness. In the example above, individuals would have a higher expected fitness if they all were doves, but doves just are not stable.

We should reiterate that while we have chosen the hawk-dove game as an example of mutual invasibility of phenotypes, any other anti-coordination game (snowdrift, chicken game, etc.) yields similar results, as will other frequency-dependent models not framed as two-player games (such as the foraging game outlined in Figure 1 of the main text). Mutually invasible phenotypes are the important thing; other details specific to the hawk-dove game are ancillary.

**III. Summary of the differences between bet-hedging and mixed ESS strategies**

Hitherto we have explored two different kinds of survival strategies in response to two different evolutionary stimuli: bet-hedging strategies in response to unpredictable environments, and stable mixed strategies in response to negative frequency dependent interactions. These two hypotheses make different experimentally testable predictions regarding the fitness dynamics between the phenotypes. The three main predictions are:

1. In a hawk-dove game, pure phenotypes are mutually invasible, while in bet-hedging they are not.
2. At the mixed ESS, all individuals in the population have the same fitness, while in a bet-hedge, each pure phenotype has a fitness advantage in different environments.
3. A bet-hedging “optimum” maximizes expected yield over time, while an evolutionarily stable strategy does not necessarily maximize yield.

### 1. Mutual invasibility

Evolutionarily stable mixed strategies arise from situations in which neither pure strategy is evolutionarily stable, because a population composed almost entirely of one phenotype can be invaded by the other, and each phenotype’s fitness is negatively correlated with its prevalence in the population. In contrast, phenotypic fitness in bet-hedging is not a function of the population composition. By probing the phenotypes for negative frequency dependence, one can establish that a hawk-dove game is likely (at least partially) responsible for the observed heterogeneity.

**
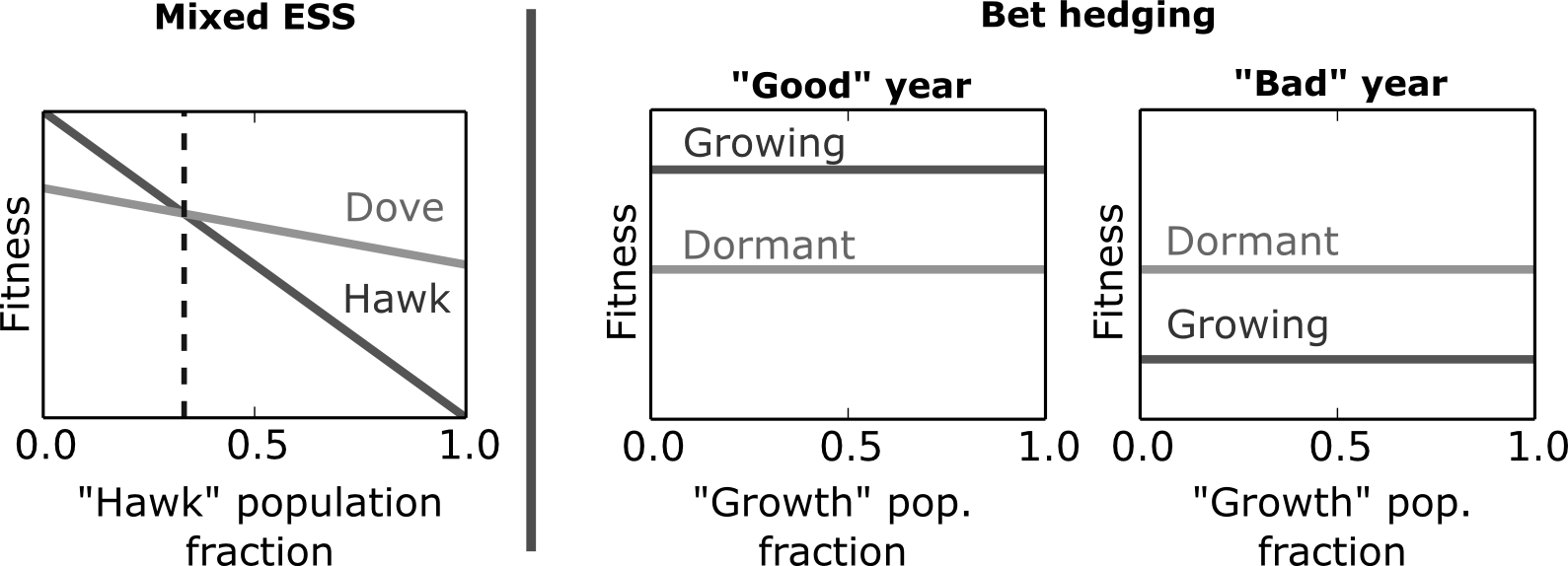
**

**Appendix Figure S14.** A side-by-side comparison of a mixed ESS (left) and a bet-hedge (right), showing fitness as a function of population frequency of each phenotype. The parameters are those previously discussed for the hawk-dove game and the seed germination examples, respectively. The cross-shaped fitness curve in the mixed ESS show the negative frequency dependence and mutual invasibility of the pure phenotypes, while the pure phenotypes in the bet-hedge are frequency-independent in both environment types.

### 2. Equal fitness of any strategy at the stable point

When a clonal population implements an evolutionarily stable mixed strategy, the phenotypic fitness of all the relevant pure strategies is equal, as is every possible mixed strategy combination of them. This is best intuited by considering the counterfactual in the context of the hawk-dove game. Suppose that there existed some other mixed strategy such that when that strategy was implemented, hawks received a higher expected payoff than doves in the population. Then it would be true that a single individual would receive a higher fitness from the “hawk” phenotype than the “dove,” and a single mutant individual which used the strategy “only play hawk” would have a higher expected fitness than the mixed strategy, and would increase in the population over time. This trend would increase the fraction of hawks in the overall population, which would in turn decrease the fitness of the hawks relative to the doves until it they were again equal. Thus, natural selection will generally drive the mixed strategy towards the point where hawks and doves have equal fitness, and, indeed where either those strategies or any mixed strategy of hawk and dove all have equal fitness. In contrast, consider the bet-hedging example from earlier. In any given environment, one of the phenotypes is more fit than the other—adopting the lower-fitness phenotype is merely a “bet” that the environment will change.

That is all well and good for a single environment, but as bet-hedging is inherently a multi-environment strategy, we may wish to make the claim that with bet hedging as with mixed strategies, all strategies are still equal *over a long period of time*, taking into account all the environmental shifts. We might reason, then, that if, *over time,* one phenotype had an advantage over the other, there would exist an incentive to alter the stochastic bet-hedging strategy to include a higher probability of the more fit phenotype, and so the optimal bet-hedging phenotype must also feature phenotypes that have equal long-term fitness.

However, this reasoning ignores an important difference between bet-hedging and mixed ESS: while it’s true that evolution will drive a bet-hedging population towards its optimal mixing frequency, this does not mean that *every* *strategy* has equal fitness, as it does in the mixed ESS. Quite the reverse: in a bet hedge, over time the optimal bet-hedge has higher fitness than any other strategy. By way of illustration, let us contrast the previous example of a mutant pure strategist hawk arising in a mixed ESS population with its counterpart in the bet-hedging example of growing and dormant phenotypes. Recall that if a mutant “pure strategist hawk” arises in a population implementing a mixed ESS, the pure strategist mutant will have *identical* *fitness* with the rest of the population, because in a mixed ESS the population fractions of each phenotype are such that hawks and doves have equal fitness. However, if a population is in an optimal bet-hedge and a mutant arises with the strategy “always remain dormant”, it is easy to see that, because it will never grow, this mutant will have lower fitness than the bet-hedging population. Likewise “always grow” will have lower fitness because it will perish in bad years. Indeed, any other strategy—randomized or otherwise--will have a lower geometric average fitness than the bet-hedging optimum. This is an essential (and experimentally verifiable) difference between mixed strategies and bet-hedges: in bet-hedging, the optimal mix of phenotypes is better than any other over time, while a population playing the mixed ESS will render a single newcomer or mutant totally indifferent to any of the possible strategies it could adopt, whether they be pure or mixed.

### 3. Not necessarily growth optimal

As we have mentioned a few times before, stable mixed strategies are unlike both bet-hedging and altruistic divisions of labor in that they are not necessarily growth-optimal for the population. This is because the optimal fitness strategies for a population often involve some sort of cooperative behavior. As an illustration, consider the *optimal* outcomes of our two games, the Prisoner’s Dilemma and the Hawk-Dove game. In both the Prisoner’s Dilemma and the Hawk-dove game, the “optimal” payoff is reached through *unstable* *pure* *strategies*: “all players cooperate” and “all players choose dove”, respectively. (They are unstable because a mutant defector or hawk can invade the population.) If the optimal average payout is instead reached through an *unstable* *mixed* *strategy* instead of a pure strategy, the unstable optimum can be considered an altruistic division of labor. Since altruistic divisions of labor are, like their pure strategy counterparts, inherently unstable, they must likewise be maintained through inclusive fitness effects like kin selection or group selection. The uninvasible strategy does not necessarily correspond to the strategy that causes the population to grow the fastest.


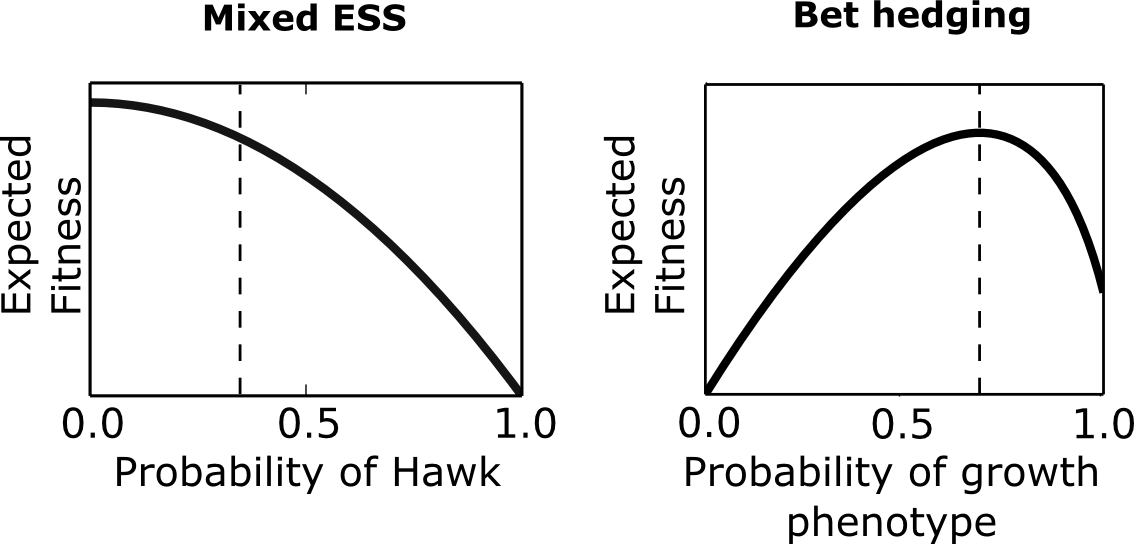


**Appendix Figure S15**. For both our mixed ESS (left) and bet hedging (right) examples, the expected fitness (of an individual or a population) is given as a function of the probability of its adopting one of the phenotypes. The parameters for the models were given previously, and fitness is measured in geometric mean fitness over time. The shape of these curves can vary (the mixed ESS curve may have mixed strategy optimum, for example). The curves serve to highlight that the bet hedging optimum is always the fitness optimum, while the mixed ESS may be sub-optimal in fitness.

**IV.**  **What if there is both frequency dependence *and* environmental uncertainty?**

Until now we have only considered “pure” cases of bet-hedging or mixed ESS. That is, cases where phenotypic heterogeneity is driven only by environmental uncertainty or only by negative frequency dependence. It is entirely possible, however, that both of those drivers may coexist within a given system, requiring a hybrid model. Whether we consider the hybrid model to be a bet-hedging model with frequency-dependent terms or a hawk-dove model with multiple possible environments, it is the same. Recall that calculating expected fitness for a bet-hedging organism in an uncertain involves taking the geometric mean of the expected fitnesses for that organism in each environment.

$$\bar{W}={[Fitness in environment G]}^{P_{G}}{[ Fitness in environment B]}^{(1-P_{G})}$$

In the example of seed germination, for instance, it may well be that fitness in the good environment is frequency dependent. Resource limitation could cause “crowding,” where germinating seeds had lower fitness as more seeds around them also germinate. The important thing to bear in mind is that frequency dependence may merely alter the optimal probabilistic bet-hedging strategy, or it may be an independent reason for heterogeneity in and of itself. In other words, if negative frequency dependence results in mutual invasibility of phenotypes (that is, both phenotypes are more fit than the other when rare), then that frequency dependence itself will stabilize phenotypic heterogeneity whether the environment is uncertain or not, and ought to be considered a stable mixed strategy.

To summarize: frequency dependent mutual invasibility alone can drive the evolution of heterogeneity, even in the absence of the environmental uncertainty, and environmental uncertainty alone can drive the evolution of phenotypic heterogeneity in the absence of mutual invasibility. If both environmental uncertainty and mutual invasibility are present in a given system, then researchers must be careful to note in their models which condition (uncertainty or frequency dependence) is driving the heterogeneity to begin with, or if both are. If the latter case, the organism could then be considered to be adopting both survival strategies simultaneously.

**Appendix Text S2: A frequency-dependent model of microbial foraging with two resources.**

In Appendix Text S1, we used a simple two player hawk-dove game to model basic predictions of scenarios with mutually invasible phenotypes. In Figure 1 of the main text, we outline a similar scenario which we call the “simple foraging game,” which we suggest would also exhibit mutual invasibility of phenotypes with a stable coexistence. In the context of microbial foraging, however, even the simple foraging game has significant conceptual deviations from the canonical two-player hawk-dove game. For example, in the hawk-dove game we generalize the payoffs of a pairwise game to an expected fitness within a population by taking an average of individual pairwise payoffs weighted by population frequencies. This works under the assumption that payoffs are linear and opponents are randomly selected. In the simple foraging game, however, it is clear than none of the interactions are pairwise to begin with, and interactions are only indirect through consumption of resources. Furthermore, during the foraging game, each microbial foraging phenotype will grow exponentially while simultaneously consuming resources, so the numbers of resources and individuals will be constantly changing nonlinearly. It is clear, then, that we need a model of mutual invasibility that is more specific to the microbial foraging game than is the canonical hawk-dove model of animal conflict.

In order, then, to better understand the dynamics of microbial foraging in multi-resourced environments, we constructed three different mathematical models of foraging. These models simulated increasingly complex versions of a foraging game, showing increasing resemblance to our model of the yeast GAL network. They are as follows: 1) the simple foraging game outlined in Figure 1 of the main text, wherein two phenotypes are available and each can only consume one of the two resources, 2) the same scenario, but in accordance with classic microbial foraging models, we allow for phenotypic switching upon depletion of one of the resources, and a corresponding diauxic lag, and 3) a game more closely approximating that played by our mutant GAL-ON and GAL-OFF pure strategists, wherein the GAL-ON mutant consumes primarily glucose even when galactose is still present.

Insights from these three simulated games include:

1. While the stable mixed strategy is the one in which both phenotypes have equal fitness, the “growth optimal” mixed strategy (defined as the mix that produces the highest average growth rate for the population) can be intuitively thought of in terms of resource consumption. The growth optimal mix is generally the phenotypic mix whose resulting population finishes both of the resources at the same time.
2. A growth rate “cost” for consuming one of the two resources is sufficient to distinguish the growth-optimal mixed strategy from the evolutionarily stable mixed strategy.
3. In several parameters—including the magnitude of the difference between the growth rates of the two phenotypes—the stable mix and the optimal mix diverge.
4. The canonical diauxic growth model, in which the resources are consumed sequentially in order of decreasing growth rate, is the unique solution (both stable and optimal) to the case in which the individuals are allowed to switch to the opposite resource with a relatively short diauxic lag time.
5. “Ratio sensing”—dividing between GAL-ON and GAL-OFF using the ratios of the two resources rather than their absolute concentrations [[23](#_ENREF_23)]—will generally either allow the cell to closely adopt the optimal or the stable mix, but not both. Which solution concept can be adopted faithfully by ratio sensing depends on the parameters of the model.

**I. The Simple foraging game**

In the basic version of the foraging game, individuals are confronted with two finite resources, $A$and *B*, and can adopt one of two corresponding mutually exclusive phenotypes, *N* and *M*. Phenotype *N* is necessary to consume resource *A*, while phenotype *M* is necessary to consume resource *B*. Growth is exponential; per capita growth is constant while corresponding resources are abundant, and zero when the resource is depleted. Table S3 lists relevant parameters. For comparability with the later simulations of the yeast glucose/galactose foraging game, simulation values for the simple general foraging game are approximated from experimental data in Appendix Figure S2 if *A* is glucose and *B* is galactose, and populations grow in a volume of 200$uL$.

Appendix Table S3. Parameters for simulation of simple foraging game

| **Parameter** | **Description** | **Default simulation value** |
| --- | --- | --- |
| $Y_{0}$ | *Initial population density* | *0.001 OD* |
| $f_{n}$ | *Starting fraction of phenotype n* | *0.8* |
| $f_{m}$ | *Starting fraction of phenotype m* | *0.2* |
| $N_{0}$ | *Initial density of phenotype n* | $f_{n}Y_{0}$ |
| $M_{0}$ | *Initial density of phenotype m* | $f_{m}Y_{0}$ |
| $A_{0}$ | *Initial density of resource A* | *0.03% (w/v)* |
| $B_{0}$ | *Initial density of resource B* | *0.05% (w/v)* |
| $r_{n}$ | *Growth rate of phenotype n on resource A* | $0.5 {hr}^{-1}$ |
| $r_{m}$ | *Growth rate of phenotype m on resource B* | $0.425 {hr}^{-1}$ |
| $\boldsymbol{\lambda}_{A}$ | *Rate of consumption of resource A by phenotype n* | $0.0003 {OD}^{-1}{hr}^{-1}$ |
| $\boldsymbol{\lambda}_{B}$ | *Rate of consumption of resource B by phenotype n* | $0.0003 {OD}^{-1}{hr}^{-1}$ |

Growth conditions are as follows:

$$\frac{\boldsymbol{dN}}{\boldsymbol{dt}}\boldsymbol{=}\left\{ \begin{aligned} r_{n}N\boldsymbol{, &A>0} \\ \boldsymbol{0, &A=0} \end{aligned} \right.$$

$$\frac{\boldsymbol{dM}}{\boldsymbol{dt}}\boldsymbol{=}\left\{ \begin{aligned} r_{m}M\boldsymbol{, &B>0} \\ \boldsymbol{0, &B=0} \end{aligned} \right.$$

$$\frac{\boldsymbol{dA}}{\boldsymbol{dt}}\boldsymbol{=}\left\{ \begin{aligned} \boldsymbol{-\lambda}_{A}N\boldsymbol{, &A>0} \\ \boldsymbol{0, &A=0} \end{aligned} \right.$$

$$\frac{\boldsymbol{dB}}{\boldsymbol{dt}}\boldsymbol{=}\left\{ \begin{aligned} \boldsymbol{-\lambda}_{B}M\boldsymbol{, &B>0} \\ \boldsymbol{0, &B=0} \end{aligned} \right.$$

Simulations were run in 1-minute increments until all the resources were consumed. Appendix Figure S16 shows an example of a single simulated run with equal starting fractions of phenotypes *n* and *m*, and with a growth cost, *c=0.15,* for consuming sugar B such that:

$$r_{m}=r_{n}\left( 1-c \right) 0\leq c\leq1$$

**
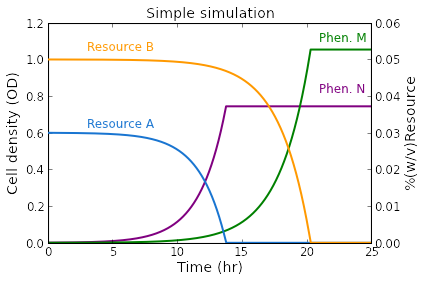
**

**Appendix Figure S16: Simple foraging game with two phenotypes and two resources**

In this simulation of a simple foraging game, phenotype N and M can consume resource A and B, respectively, but not the opposite resource. Resource B is more abundant than resource A, but consuming resource B incurs a 15% growth cost. Phenotypes N and M start out at a ratio of 80/20 in the population.

It is important to note that in the simulations, phenotype *m* consumes resources at the same rate as phenotype n, but it has a smaller growth rate. This simulates the presumably common scenario wherein a proportion of the energy extracted from a food source goes towards paying the metabolic cost of producing the proteins and enzymes necessary to consume the resource.

We next investigated the frequency-dependent game played between individuals of the two metabolic phenotypes. By running the simulation with a range of different starting fractions of phenotype *m,* $f_{m}$, we show that even in this very simplified foraging game, our experimental results are borne out in simulation. There is strong negative frequency dependence and mutual invasibility between the phenotypes (Appendix Figure S17 a-b), with an intermediate stable equilibrium at about 60% phenotype *m.*  We also calculated an average growth rate for the population defined as the final population density (which is basically the same regardless of population composition) divided by the time it took the population to reach saturation. We found, as we did with our experimental results, that the “optimal mix”—the composition that maximizes the population’s growth—contains much more phenotype *m* than the evolutionarily stable mix (Appendix Figure S17, grey dashed line). Since the final population densities are equal (Appendix Figure S17), average growth rate can be thought of as mostly a function of the time it takes a population to consume all of the sugars. The optimal mix, with higher proportion of phenotype *m,* finishes the sugars the fastest, and is also the one that finishes the sugars at the same time. Indeed, in all cases where phenotype *m* has lower growth rate than phenotype *n,* we find the optimal mix contains a higher proportion of phenotype *m* than does the stable mix.

This model can also be solved analytically by noting that until resource A is exhausted we have:

$$\frac{dN}{dt}=r_{n}N=-\frac{r_{n}}{\lambda_{A}}\frac{dA}{dt}$$

Integration then yields:

$$N_{f}-N_{0}= -\frac{r_{n}}{\lambda_{A}}\left( A_{f}-A_{0} \right)=\frac{r_{n}}{\lambda_{A}}A_{0}$$

where we have assumed that the final number of the *N* type (*N_f_*) occurs when resource A is equal to zero. Similarly,

$$M_{f}-M_{0}=\frac{r_{n}\left( 1-c \right)}{\lambda_{B}}B_{0}$$

The equilibrium occurs when the *N* type and *M* type have the same number of doublings. In particular, if we perform multiple cycles of dilution and growth (to saturation) with dilution factor *D* then at equilibrium:

$$D=\frac{N_{f}}{N_{0}}=1+\frac{r_{n}}{\lambda_{A}N_{0}}A_{0}$$

$$D=\frac{M_{f}}{M_{0}}=1+\frac{r_{n}\left( 1-c \right)}{\lambda_{B}M_{0}}B_{0}$$

The equilibrium ratio of phenotype *N* to phenotype *M* is therefore:

$$\frac{N_{0}}{M_{0}}=\frac{A_{0}\lambda_{B}}{B_{0}\left( 1-c \right)\lambda_{A}}$$

**
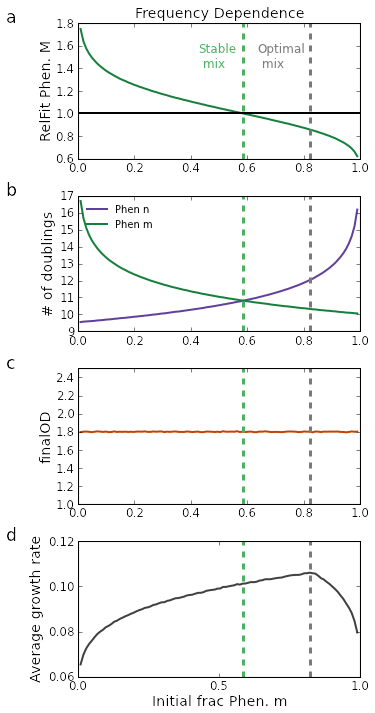
**

**Appendix Figure S17. Frequency dependence of simple foraging game**

As a function of initial population frequency of *m* phenotype: ***a*)** Relative fitness of the *m* phenotype and **b)** absolute fitness of both *n* (purple) and *m* (green) phenotypes indicate negative frequency dependence and mutual invasibility between phenotypes. Each phenotype is more fit than the other when rare. **c)** The final total population density remains essentially unchanged by the phenotypic composition of the population. However, since the time to reach the final OD does change depending on the composition of the population, then **(d)** the average growth rate of the population is maximized at an intermediate frequencies. However, the optimal mix for the population (grey dotted line) is not the same as the evolutionarily stable mix (green dotted line).

### Divergence of stable and optimal mixes

We next investigated the stable and optimal mixes as a function of phenotypic growth rates. Unsurprisingly, both the stable and the optimal mixes changed depending on the growth disparity between the phenotypes, but remarkably, they changed in opposite directions. As phenotype *m* gets less and less fit relative to phenotype *n,* the evolutionarily stable strategy is to adopt phenotype *m* less frequently, while the growth optimal strategy is to adopt phenotype *m* more frequently. This difference can be understood intuitively by recalling the informal definitions of the stable and the optimal mix.

The stable mix is the one in which all individuals have the same fitness regardless of phenotype. Hence, with negative frequency dependent fitness, if phenotype m becomes less fit, more individuals must adopt phenotype *n* until their fitness is correspondingly lower. On the other hand, the growth optimal mix is the one in which the two resources are consumed at the same time (so that no phenotype sits idle). A lower fitness for phenotype *m* means that resource *B* will take longer to consume than resource *A*, so to maximize the growth of the population, individuals should adopt phenotype *m* to consume resource *B* more quickly. Appendix Figure S18 illustrates the divergence of the stable and the optimal mixes as a function of the fitness cost of phenotype *m.*  Intriguingly, although the “optimal mix” population grows much faster than the “stable mix” population, an evolutionary competition between the two mixed strategies in the absence of opportunities for group or kin selection would favor would favor the slower-growing stable mix. A spatially-structured environment, however, may favor the optimal mix through group or kin selection[[24](#_ENREF_24)]. The simulations demonstrate that the two solution concepts diverge even with a relatively simple two-resource scenario.

**
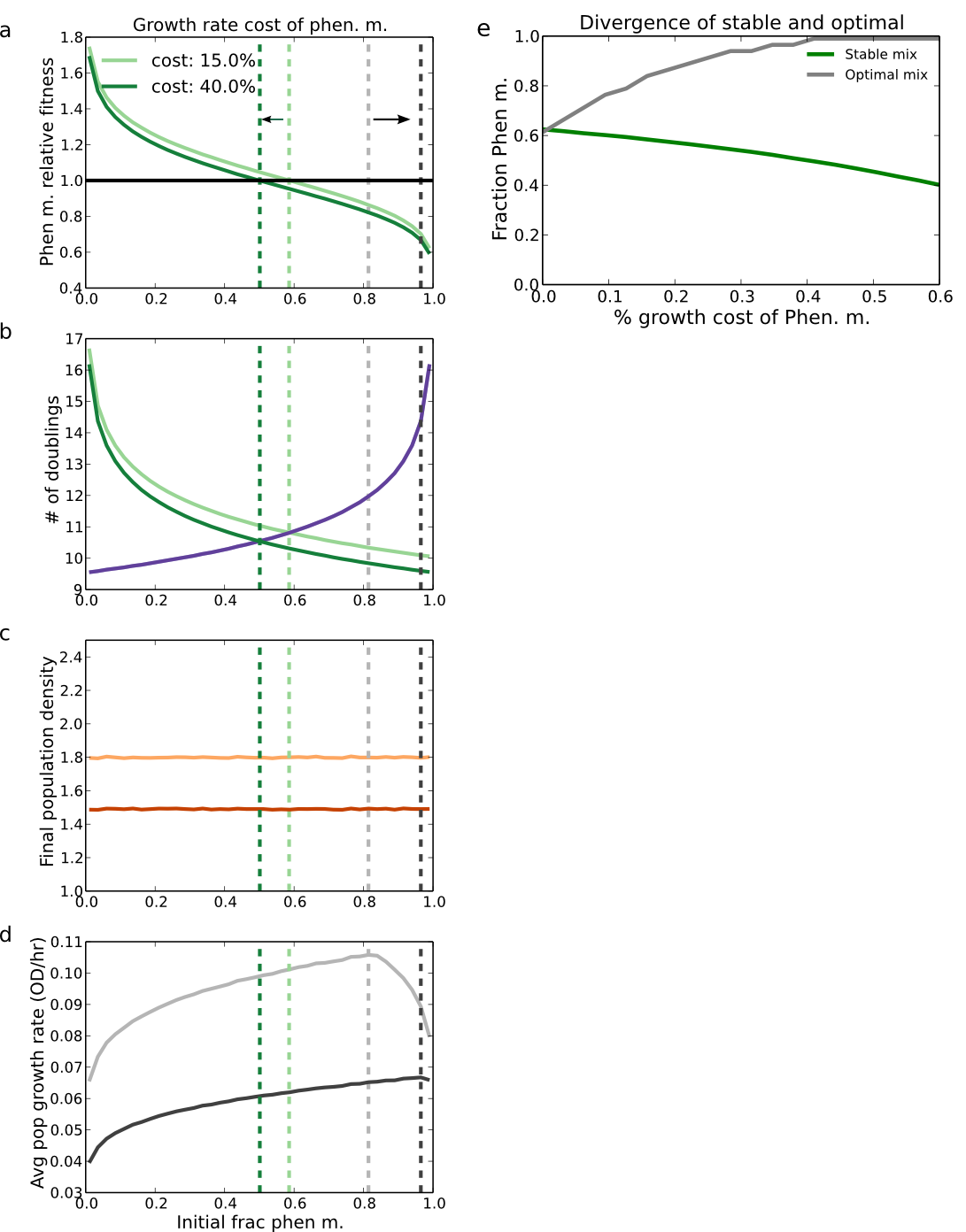
**

**Appendix Figure S18:** **Stable and optimal mixes diverge with increasing metabolic cost of phenotype *m.***

**(a-d)** Frequency dependent fitness, similar to Figure 2, is plotted for two different growth costs of adopting phenotype *m*: *c = 0.15%* (light) and *c=0.4%* (dark)*.* In other words, in the light case, phenotype *m* grows 15% slower and in the dark case it grows 40% slower than phenotype *n.* Higher phenotype *m* growth cost lowers the total number of divisions possible on resource B, shifting the stable equilibrium left, while the growth optimal mix (**d,** grey dotted lines) shifts right. **e)** The stable mix (green) and optimal mix (gray) are plotted as a function of the growth cost of adopting phenotype *m.* The fact that the two solution concepts not only are different but also diverge serves to highlight that mixed ESS and optimal divisions of labor are very different solution concepts.

## II. Foraging game with facultative phenotypic switching

The simple foraging game outlined above assumes that each phenotype can only consume one of the resources exclusively. However, this assumption is bears little resemblance to most microbial environments with multiple carbon sources. In reality, individuals can generally sense the presence of a resource and adopt the appropriate phenotype for consuming it, switching from one to the other if necessary. Indeed, the canonical response to multiple sugars is thought to be a diauxic response: consume the most advantageous carbon source first, then switch to the next most advantageous, and so on. A reasonable follow-up to the simple game, therefore, is to relax the consumption constraints and allow individuals to switch their phenotype when the resource runs out. However, given that many metabolic regimes such as galactose or lactose consumption involve the production of large numbers of specialized metabolic enzymes, a diauxic lag phase is typically observed during the switch. Given that little growth happens during this time, such a lag phase represents a cost to switching. Accordingly, for our simulations, we altered the simple foraging simulation to allow such phenotypic switching, and we introduced a new parameter, θ, the diauxic lag time.

Growth therefore occurs in three phases. Before any resources are exhausted we have:

$$\frac{dN}{dt}=r_{n}N$$

$$\frac{dM}{dt}=r_{m}M$$

$$\frac{dA}{dt}=-\lambda_{A}N$$

$$\frac{dB}{dt}=-\lambda_{B}M$$

Assuming that it is resource A that is first exhausted, for a time θ we have:

$$\frac{dN}{dt}=0$$

$$\frac{dM}{dt}=r_{m}M$$

$$\frac{dB}{dt}=-\lambda_{B}N$$

After this diauxic lag period both cell types then grow on resource B according to:

$$\frac{dN}{dt}=r_{m}N$$

$$\frac{dM}{dt}=r_{m}M$$

$$\frac{dB}{dt}=-\lambda_{A}N-\lambda_{B}M$$

| **Parameter** | **Description** | **Default simulation value** |
| --- | --- | --- |
| $\theta$ | *Diauxic shift lag time* | *2.0 hr* |

Appendix Figure S19 shows simulations with the same default growth parameters as in Appendix Figure S16, but with ability to switch phenotypes after sugars are consumed and with a lag growth time of 2 hrs.


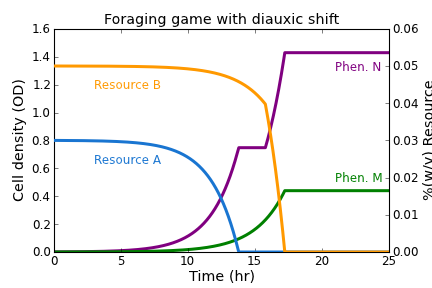


**Appendix Figure S19:** **A simulated foraging game allowing for diauxic growth with a diauxic lag time of 2 hr.** Individuals of phenotypes *n* and *m* begin at 80% and 20% of the population, respectively, and consume resource A and resource B, respectively. At around 13hr, resource A is depleted and individuals of phenotype N switch to consuming resource B after a diauxic lag of 2 hours.

### Diauxic lag, stability, and optimality

To explore the effects of the switching costs on the stable mixed and optimal mixed strategies, we ran simulations with many different switching costs. As expected, at the limit of high diauxic lag time—or very costly switching—frequency dependence is identical to that in the simple foraging game (Appendix Figure S20a, darkest curve). At the limit of very low diauxic lag time, frequency dependence vanishes entirely (Appendix Figure S20a, lightest curve), and phenotype *n* is more fit than phenotype *m* regardless of population composition. Below a threshold lag time, the optimal and stable solution to the foraging game is the classic diauxic growth strategy: all individuals consume the best resource first.

With intermediate diauxic lag times, the situation is somewhat more complicated. The relative fitness of phenotype *m* as a function of its population frequency can be essentially divided into three sections: low, intermediate, and high fm. At intermediate $f_{m}$—close to the optimal mix—the fitness of the two phenotypes are identical to those in the simple foraging game (Appendix Figure S20a) because the two resources are consumed close enough together that there is not time enough for the faster phenotype to switch before the other resource is gone also. The higher the diauxic lag time, the wider this intermediate fitness zone is. One corollary of this observation is that, since the optimal mix is the one in which both resources are consumed at the same time, the optimal mix is not really responsive to differences in the diauxic lag time (Appendix Figure S20d). And in the parameters we have outlined, the stable mix follows a similar pattern, though there is a remarkable phenomenon where in low-to-intermediate lag times (1.5-2.0 hr in Appendix Figure S20d) the population mix that grows the fastest consists almost entirely of phenotype *m*, while the population mix that is evolutionarily stable is 100% phenotype *n.*

**
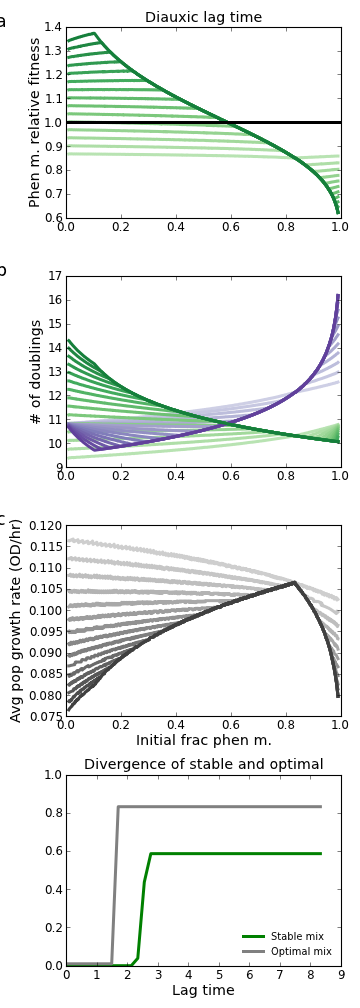
**

**Appendix Figure S20 The effect of diauxic lag times on the stable and optimal solutions to the foraging game**

Frequency dependent **a)** relative fitness, **b)** number of divisions, and **c)** average population growth are shown for a range of diauxic lag times from 0 to 8 hours. At high diauxic lag times, the frequency dependence is identical to the simple model simulation, and at the limit of low diauxic lag times, frequency dependence disappears altogether and phenotype *n* is more fit than *m* at all frequencies. **d)** The stable (green) and optimal (gray) mixed strategies are shown as a function of diauxic lag time. Both solutions display transition between no phenotype *m* at low lag times to intermediate frequencies of phenotype *m* at high lag times.

## III. Simulation of GAL-OFF and GAL-ON pure strategists

To try to better understand the foraging game being played between our GAL-ON and GAL-OFF pure strategist strains, we undertook a simulation that roughly approximates our understanding of that game. Because the GAL-OFF pure strategists do not switch to GAL-ON during the course of the experiment, the game resembles the simple foraging game, with the exception that in this case, phenotype *m* (the GAL-ON phenotype) can consume resource *A* (glucose) even when resource *B* is abundant. This necessitates us replacing the $r_{m}$ and parameter with sugar-specific parameters.

| **Parameter** | **Description** | **Default simulation value** |
| --- | --- | --- |
| $r_{m}^{A}$ | *GAL-ON growth rate on glucose* | $0.425 {hr}^{-1}$ |
| $r_{m}^{B}$ | *GAL-ON growth rate on galactose* | $0.3825 {hr}^{-1}$ |
|  |  |  |

Because in the mixed sugar conditions the GAL-ON cells are still GAL-ON even when consuming glucose, their growth rate on glucose reflects the 15% growth cost we imputed to them earlier. GAL-ON’s growth rate on galactose was also estimated from experimental values. The most salient question that arose during these simulations was the amount of galactose that GAL-ON pure strategists consume. Some researchers have claimed that, in the presence of substantial glucose, even GAL-activated yeast do not consume substantial amounts of galactose [[25](#_ENREF_25)], while others have observed some galactose consumption [[23](#_ENREF_23),[26](#_ENREF_26)]. Our own data is somewhat ambiguous (see Appendix Figure S2). It appears that before the glucose is entirely consumed, the galactose has been somewhat consumed, though not to the same degree. Under growth parameters estimated from our experimental data, in order to see stable mixed strategies of intermediate frequencies we must either make the GAL network more costly to run than we anticipated (15-30% growth cost) or allow the GAL-ON mutants to consume significant galactose even in the presence of glucose (in that case, we assume that galactose is displacing rather than adding to the glucose in the metabolic pathways). Appendix Figure S21 illustrates the frequency-dependent fitness as a function of the proportion of GAL-ON’s sugar consumption being galactose.

**
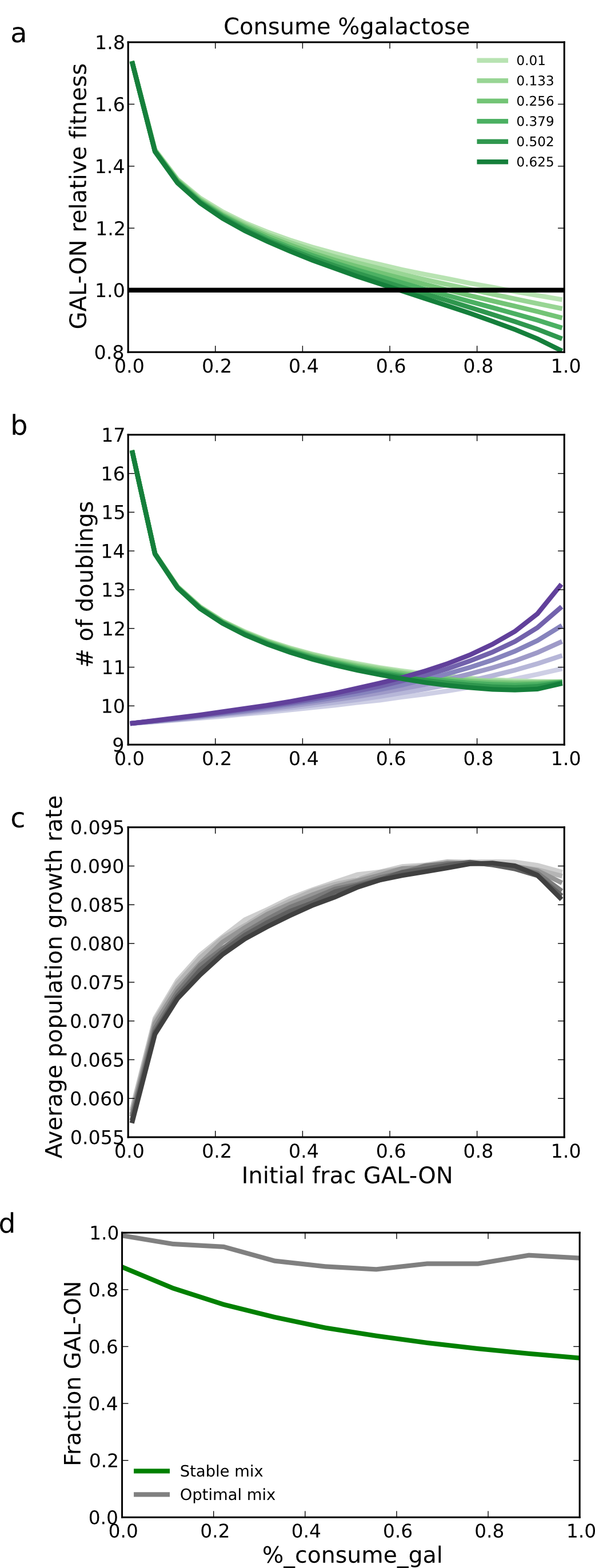
**

**Appendix Figure S21. GAL-ON and GAL-OFF frequency dependence in .03/.05% glu/gal** **as a function of the distribution of sugar uptake for GAL-ON in mixed sugars.**

Frequency dependent **a)** relative fitness, **b)** number of divisions, and **c)** average population growth are shown for a range different galactose-consumption conditions, ranging from GAL-ON only consuming 1% galactose while glucose is still present (lightest curve) to GAL-ON’s consuming galactose in proportion to its initial fraction of total sugars (62.5%, darkest curve). The amount of galactose that GAL-ON consumes in the presence of glucose does not have a large effect on the optimal mix **(c,d),** but does have a large effect on the stable mix **(a,d) d)** The stable (red) and optimal (green) mixed strategies are shown as a function of the proportion of galactose consumed in the presence of both glucose and galactose.

**IV. Stochastic switching**

Unlike the GAL-OFF and GAL-ON pure strategies, the wildtype yeast cells are able to stochastically switch between the GAL ON and GAL OFF states. If this switching is sufficiently fast then it is expected to “wash” out the frequency dependence that is observed between cells that start out ON versus OFF. To explore the consequences of this stochastic switching for the frequency dependence observed in our simple model (Fig S16), we performed simulations of a stochastic model that allows for switching between these states with switching rates that are dependent upon the sugar concentration, as follows:

$$\frac{dN_{OFF}}{dt}=\gamma_{\max}\frac{[gluc]}{\left[ gluc \right]+K_{gluc}}N_{OFF}-k_{ON}N_{OFF}+k_{OFF}N_{ON}$$

$$\frac{dN_{ON}}{dt}=\gamma_{\max}\left( 1-c \right)\max\left( \frac{\left[ gluc \right]}{\left[ gluc \right]+K_{gluc}},\frac{\left[ gal \right]}{\left[ gal \right]+K_{gal}} \right)N_{ON}+k_{ON}N_{OFF}-k_{OFF}N_{ON}$$

In these equations we are assuming that the ON cells must pay the metabolic cost c of producing the galactose genes but is able to choose between consuming glucose or galactose to maximize growth (we found that through most of the growth cycle the GAL ON cells are consuming primarily glucose).

The sugars are then assumed to be exhausted according to:

$$\frac{d\left[ gluc \right]}{dt}={- \beta\frac{dN_{OFF}}{dt}}$$

$$\frac{d[gal]}{dt}={- \beta\frac{dN_{ON}}{dt}}$$

if the ON cells are consuming galactose and

$$\frac{d\left[ gluc \right]}{dt}={- \beta\left( \frac{dN_{OFF}}{dt}+\frac{dN_{ON}}{dt} \right)}$$

$$\frac{d[gal]}{dt}=0$$

if the ON cells are not consuming galactose (ie if more rapid growth can be attained by consuming glucose.

$$\gamma_{\max}=0.5 hr^{-1}$$

$$K_{gluc}=0.005\%$$

$$K_{gal}=0.02\%$$

$$k_{on}=0 \text{for the no switching condition}$$

$$k_{on}=\left( 0.5 hr^{-1} \right)\frac{\left[ gal \right]}{\left[ gal \right]+C\left[ gluc \right]}$$

$$C=\left( 125,50 \right) \text{for the intermediate and rapid switching conditions}$$

$$k_{off}=\left( 0, 0.01,0.1 \right)hr^{-1} \text{for the no, intermediate, and rapid switching conditions}$$

$$\beta=0.05$$

$$c=0.1$$

To measure frequency dependence, we initiated populations with a total optical density of 0.004 and various starting fractions of ON and OFF. We then allowed both populations to divide and switch states, but kept track of the lineages that started from the GAL ON and GAL OFF states. Consistent with the predictions from our simpler model, we see that there is negative frequency dependent selection in this model in the absence of switching (dark grey is for population starting GAL OFF, light grey is for population starting GAL ON). As we increase the rate of switching, we see that the strength of the negative frequency dependence decreases. This is because as the two competing populations begin to switch they become more similar to one another. Indeed, as the switching rate becomes very rapid the two populations are both similarly mixed as the glucose becomes exhausted and negative frequency dependence disappears (Fig S22).

Our experiments with the wildtype yeast strains indicate that the sorted populations starting all ON or all OFF retain some memory of their initial state at the time that the glucose becomes exhausted, consistent with the negative frequency dependence that we observe. Interestingly, the stochastic switching rates are a function of the sugar concentrations (less switching at higher sugar concentrations), meaning that the strength of negative frequency dependence in this system will also depend upon the sugar concentrations.


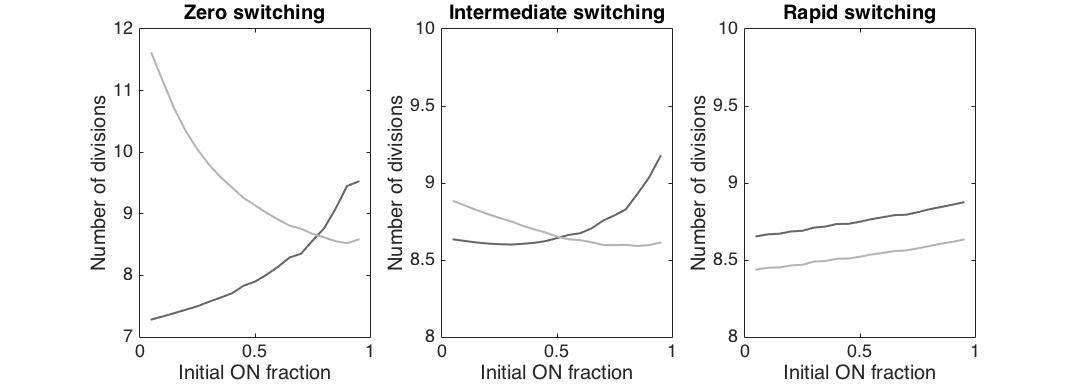


**Appendix Figure S22. The effect of phenotypic switching rate on mutual invasibility**. Fitness (in number of divisions) is shown for simulations of competition between cells that are initially GAL-ON and cells that are initially GAL-OFF. The cells are competed in finite mixed glucose and galactose, with sugar depletion, and allowing for no switching (left), intermediate (middle) and rapid (right) stochastic switching. Switching rates from the ON to the OFF state are 0, 0.01, and 0.1 hr^-1^ respectively, whereas the switching rates from the OFF to the ON state depend upon the sugar concentration for the intermediate and rapid switching cases (for the no switching case the cells are unable to switch in either direction). As switching rate increases, the initial GAL-ON and GAL-OFF populations resemble each other more closely for more of the course of the simulation. Thus, higher stochastic switching rate decreases the magnitude of the frequency dependence.

**References for Supporting Information**

1. Slatkin M (1974) Hedging Ones Evolutionary Bets. Nature 250: 704-705.

2. Veening JW, Smits WK, Kuipers OP (2008) Bistability, epigenetics, and bet-hedging in bacteria. Annu Rev Microbiol 62: 193-210.

3. Muller J, Hense BA, Fuchs TM, Utz M, Potzsche C (2013) Bet-hedging in stochastically switching environments. J Theor Biol 336: 144-157.

4. McNamara JM (1997) Optimal life histories for structured populations in fluctuating environments. Theoretical Population Biology 51: 94-108.

5. Cohen D (1966) Optimizing Reproduction in a Randomly Varying Environment. Journal of Theoretical Biology 12: 119-&.

6. Cohen D (1967) Optimizing Reproduction in a Randomly Varying Environment When a Correlation May Exist between Conditions at Time a Choice Has to Be Made and Subsequent Outcome. Journal of Theoretical Biology 16: 1-&.

7. Gremer JR, Venable DL (2014) Bet hedging in desert winter annual plants: optimal germination strategies in a variable environment. Ecol Lett.

8. Philippi T (1993) Bet-hedging germination of desert annuals: beyond the first year. Am Nat 142: 474-487.

9. Lennon JT, Jones SE (2011) Microbial seed banks: the ecological and evolutionary implications of dormancy. Nature Reviews Microbiology 9: 119-130.

10. Clauss MJ, Venable DL (2000) Seed Germination in Desert Annuals: An Empirical Test of Adaptive Bet Hedging. Am Nat 155: 168-186.

11. Gamelon M, Gaillard JM, Baubet E, Devillard S, Say L, et al. (2013) The relationship between phenotypic variation among offspring and mother body mass in wild boar: evidence of coin-flipping? Journal of Animal Ecology 82: 937-944.

12. Evans ML, Dionne M, Miller KM, Bernatchez L (2012) Mate choice for major histocompatibility complex genetic divergence as a bet-hedging strategy in the Atlantic salmon (Salmo salar). Proceedings of the Royal Society B-Biological Sciences 279: 379-386.

13. Balaban NQ, Merrin J, Chait R, Kowalik L, Leibler S (2004) Bacterial persistence as a phenotypic switch. Science 305: 1622-1625.

14. Kussell E, Kishony R, Balaban NQ, Leibler S (2005) Bacterial persistence: a model of survival in changing environments. Genetics 169: 1807-1814.

15. Levy SF, Ziv N, Siegal ML (2012) Bet hedging in yeast by heterogeneous, age-correlated expression of a stress protectant. PLoS Biol 10: e1001325.

16. Veening JW, Stewart EJ, Berngruber TW, Taddei F, Kuipers OP, et al. (2008) Bet-hedging and epigenetic inheritance in bacterial cell development. Proceedings of the National Academy of Sciences of the United States of America 105: 4393-4398.

17. Solopova A, van Gestel J, Weissing FJ, Bachmann H, Teusink B, et al. (2014) Bet-hedging during bacterial diauxic shift. Proc Natl Acad Sci U S A.

18. Jones SE, Lennon JT (2010) Dormancy contributes to the maintenance of microbial diversity. Proceedings of the National Academy of Sciences of the United States of America 107: 5881-5886.

19. Piggot P (2010) Epigenetic Switching: Bacteria Hedge Bets about Staying or Moving. Current Biology 20: R480-R482.

20. Stumpf MP, Laidlaw Z, Jansen VA (2002) Herpes viruses hedge their bets. Proc Natl Acad Sci U S A 99: 15234-15237.

21. Meadows R (2012) Yeast survive by hedging their bets. PLoS Biol 10: e1001327.

22. de Jong IG, Haccou P, Kuipers OP (2011) Bet hedging or not? A guide to proper classification of microbial survival strategies. Bioessays 33: 215-223.

23. Escalante-Chong R, Savir Y, Carroll SM, Ingraham JB, Wang J, et al. (2015) Galactose metabolic genes in yeast respond to a ratio of galactose and glucose. Proc Natl Acad Sci U S A 112: 1636-1641.

24. Nowak MA (2006) Five rules for the evolution of cooperation. Science 314: 1560-1563.

25. Venturelli OS, Zuleta I, Murray RM, El-Samad H (2014) Population diversification in a yeast metabolic program promotes anticipation of environmental shifts. bioRxiv.

26. Wang J, Atolia E, Hua B, Savir Y, Escalante-Chong R, et al. (2015) Natural variation in preparation for nutrient depletion reveals a cost-benefit tradeoff. PLoS Biol 13: e1002041.

1. If, instead, $C<\frac{1}{2}V$, then the game becomes a Prisoner’s Dilemma with Hawk being the only stable strategy. [↑](#footnote-ref-1)
